# The tumor suppressor *MIR139* is silenced by POLR2M to promote AML oncogenesis

**DOI:** 10.1038/s41375-021-01461-5

**Published:** 2021-11-05

**Authors:** Christiaan J. Stavast, Iris van Zuijen, Elena Karkoulia, Arman Özçelik, Antoinette van Hoven-Beijen, Leticia G. Leon, Jane S. A. Voerman, George M. C. Janssen, Peter A. van Veelen, Monika Burocziova, Rutger W. W. Brouwer, Wilfred F. J. van IJcken, Alex Maas, Eric M. Bindels, Vincent H. J. van der Velden, Christopher Schliehe, Peter D. Katsikis, Meritxell Alberich-Jorda, Stefan J. Erkeland

**Affiliations:** 1 Erasmus MC, University Medical Center Rotterdam, Department of Immunology, Rotterdam, the Netherlands; 2 Department of Hemato-Oncology, Institute of Molecular Genetics of the Czech Academy of Sciences, Prague, Czech Republic; 3 Childhood Leukemia Investigation Prague, Department of Pediatric Haematology and Oncology, 2nd Faculty of Medicine, Charles University in Prague, Prague, Czech Republic; 4 Center for Proteomics and Metabolomics, Leiden University Medical Center, Leiden, the Netherlands; 5 Erasmus MC, University Medical Center Rotterdam, Center for Biomics, Rotterdam, the Netherlands; 6 Erasmus MC, University Medical Center Rotterdam, Department of Cell Biology, Rotterdam, the Netherlands; 7 Erasmus MC, University Medical Center Rotterdam, Department of Hematology, Rotterdam, the Netherlands

**Keywords:** Acute myeloid leukaemia, Cancer genetics, Apoptosis, Oncogenes

## Abstract

*MIR139* is a tumor suppressor and is commonly silenced in acute myeloid leukemia (AML). However, the tumor-suppressing activities of *miR-139* and molecular mechanisms of *MIR139*-silencing remain largely unknown. Here, we studied the poorly prognostic MLL-AF9 fusion protein-expressing AML. We show that MLL-AF9 expression in hematopoietic precursors caused epigenetic silencing of *MIR139*, whereas overexpression of *MIR139* inhibited in vitro and in vivo AML outgrowth. We identified novel *miR-139* targets that mediate the tumor-suppressing activities of *miR-139* in MLL-AF9 AML. We revealed that two enhancer regions control *MIR139* expression and found that the polycomb repressive complex 2 (PRC2) downstream of MLL-AF9 epigenetically silenced *MIR139* in AML. Finally, a genome-wide CRISPR-Cas9 knockout screen revealed RNA Polymerase 2 Subunit M (POLR2M) as a novel *MIR139*-regulatory factor. Our findings elucidate the molecular control of tumor suppressor *MIR139* and reveal a role for *POLR2M* in the *MIR139*-silencing mechanism, downstream of MLL-AF9 and PRC2 in AML. In addition, we confirmed these findings in human AML cell lines with different oncogenic aberrations, suggesting that this is a more common oncogenic mechanism in AML. Our results may pave the way for new targeted therapy in AML.

## Introduction

Mixed-lineage leukemia (MLL) or Lysine Methyltransferase 2A (KMT2A) rearrangements occur in 10% of adult acute leukemias and in 35% of pediatric cases [1, 2]. MLL rearrangements cause in-frame fusions with >60 different genes of which *AF4*, *AF9*, *AF10*, *ENL,* and *ELL* are most common [3, 4]. MLL-fusion proteins cause heterogeneous leukemia of lymphoid or myeloid origin and are associated with therapy resistance and poor prognosis [3, 5]. MLL-fusion proteins retain the DNA-binding domain of MLL and deregulate target genes that are normally controlled by MLL [6]. The most common MLL-rearrangement in acute myeloid leukemia (AML) is caused by the t(9;11)(p22q23) fusing *MLL* to the *AF9* gene in-frame and occurs in 30% of MLL-rearranged AMLs [3, 5]. Continuous expression of MLL-AF9 is critical for the survival of AML cells, which suggests that genes deregulated by MLL-AF9 are potential targets for therapy [7, 8].

MicroRNAs (miRNAs) are small non-coding RNAs of 19–22 nt in length and post-transcriptionally regulate mRNAs involved in critical cellular functions [9]. MiRNAs are commonly deregulated in cancer [10, 11]. For instance, the expression of *MIR139*, encoding *miR-139-5p* and *miR-139-3p*, is downregulated and has been identified as a tumor suppressor in AML [12–14]. The *MIR139* gene is located on chromosome-11 in intron-1 of *PDE2A* [15]. However, the molecular mechanism of *MIR139* silencing in AML is largely unknown. Previously, we have found that *miR-139-3p* expression is induced by DNA interstrand-crosslinks [12]. There is evidence that *miR-139-5p* controls the translation machinery through downregulation of *Eif4g2* [13, 14].

Polycomb-group (PcG) proteins have been implicated in silencing tumor suppressor genes in various cancers, including MLL-AF9 AML [16–19]. One class of PcG proteins, polycomb repressive complex 2 (PRC2), consists of the methyltransferase EZH2 or EZH1, EED, SUZ12, and RBBP4 [20]. EZH2 or EZH1 can hypermethylate K27 on Histone-H3, which marks silenced genes [21]. PRC2 contributes to MLL-AF9 pathogenesis and is a target for therapy [22, 23]. However, an additional unknown activity mediated by PRC2 that regulates transcriptional repression, other than blocking the accessibility of chromatin remodeling proteins, cannot be ruled out [24].

Here, we have performed a comprehensive analysis of the tumor suppressor *MIR139*. We show that *MIR139* is a critical tumor suppressor that is silenced by MLL-AF9 in AML. Furthermore, we have identified *MIR139* targets that mediate its tumor suppressor activity in AML. Our study has elucidated the molecular silencing mechanism of *MIR139* and provides insight into its tumor suppressor activity, offering potential targets of intervention for the poorly prognostic MLL-AF9 AML.

## Methods

### Generation of knockout mice

C57BL/6 *Mir139*-deficient mice (*Mir139*KO) were generated using the CRISPR-Cas9 double-nicking strategy employed by the Cas9^D10A^ mutant and 4 sgRNAs (Mir139KOsgRNA5’plus, Mir139KOsgRNA5’minus, Mir139KOsgRNA3’plus, and Mir139KOsgRNA3’minus) flanking the *Mir139* gene as described [25]. Enhancer 1 KO mice (E1KO) and E2KO mice were generated by zygote injection with TrueCut Cas9 Protein v2 (A36499, Thermo Fisher Scientific) and sgRNAs E1KO-5p, E1KO-3p, E2KO-5p, and E2KO-3p (Supplementary Table 1). Heterozygous or homozygous offspring were crossed for at least three generations to reduce CRISPR-Cas9-induced off-target effects. All primers and sgRNAs are listed in Supplementary Table 1. All animal experiments were approved by the Instantie voor Dierenwelzijn (IvD) and the Erasmus Medical Center Animal Welfare and Ethics Committee.

### Colony assays, inhibitor experiments, cell culture, and patient samples

For colony assays, 5000 MLL-AF9 cells were plated in MethoCult (M3231, StemCell Technologies), supplemented with IL-3 (1:1000 supernatant), iIL-6 (10 ng/mL), granulocyte-macrophage colony-stimulating factor (GM-CSF) (10 ng/mL), and stem cell factor (SCF) (20 ng/mL), penicillin/streptomycin (100 U/mL) and neomycin 500 µg/mL and expanded for 7 days. DOT1L inhibitor SGC0946 (SML1107, Sigma Aldrich) or UNC1999 (SML0778, Sigma Aldrich) were dissolved in dimethyl sulfoxide (DMSO) and added to the medium at indicated concentrations. Human MLL-AF9 patients were diagnosed according to WHO criteria by cytomorphology, cytogenetics, molecular diagnostics and flow cytometric immunophenotyping. Mononuclear cells were isolated by Ficoll-Paque (density 1.077 g/mL) from peripheral blood or bone marrow. All included patients were positive for the t(9;11)(p22;q23) translocation and samples used for this study contained at least 84% AML blasts as defined by flow cytometry [26]. The use of human MLL-AF9 patient samples was approved by the Erasmus MC Medical Ethics Committee (METC 2016-606). Human MLL-AF9 AML cell lines THP-1, Molm-13, and other AML cell lines MV4-11, HL-60, HEL, and U937 were cultured in Roswell Park Memorial Institute-1640 supplemented with Pen-Strep (100 U/mL), fetal bovine serum (FBS) (10%), and l-Glut (2 mM). AML cell line Kasumi-1 was cultured as above with 20% FBS and AML cell line TF-1 was cultured as above supplemented with human granulocyte-macrophage colony-stimulating factor (5 ng/mL, 300-03-B, Peprotech) and sodium pyruvate (1%). The MLL-AF9 patient samples were cultured in Iscove’s Modified Dulbecco’s Medium, supplemented with FBS (20%), Pen-Strep (100 U/mL), IL-3 (10 ng/mL, 200-03, Peprotech), IL-6 (10 ng/mL, 200-06, Peprotech), TPO (50 ng/mL, 300-18, Peprotech), FLT-3L (50 ng/mL, 300-09, Peprotech), and SCF (50 ng/mL, 300-07, Peprotech).

### Statistical analyses

Data are presented as mean ± SEM, unless otherwise specified in the figure legends. Data were compared with the two-tailed independent samples Student’s *t* test, unless otherwise specified in the figure legends.

## Results

### MLL-AF9 downregulates *Mir139* expression

**Fig. 1:**
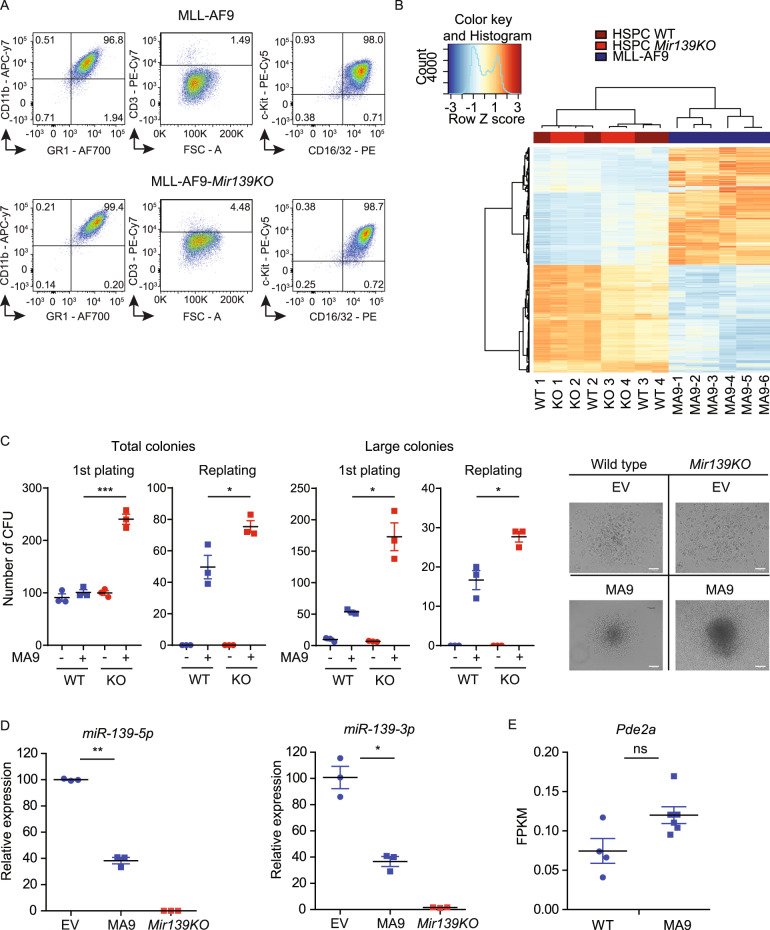
MLL-AF9 downregulates *Mir139* expression. **A** Flow cytometric analysis of surface marker expression of MLL-AF9 or MLL-AF9-*Mir139*KO cells stained for myeloid lineage markers CD11b, CD16/32, c-Kit, GR-1 and lymphoid lineage marker CD3. **B** Heatmap showing the differential gene expression profiles of WT HSPCs (WT), *Mir139*KO HSPCs (KO), and MLL-AF9 cells (MA9). **C** The number of colony-forming units (CFUs) per 5000 empty vector control (−) and MLL-AF9 (MA9) transduced WT and *Mir139*KO HSPCs are shown. Total number of colonies (50–250 cells/colony) and large colonies (>250 cells/colony) are presented. Micrographs depict representative colonies. Scale bar indicates 100 µm. The two-tailed unpaired student’s *t* test was used for the statistical analysis. **D** Expression of *miR-139-5p* and *miR-139-3p* in MA9 cells and MLL-AF9-*Mir139*KO relative to snRNA U6 and EV transduced HSPCs (EV) as determined by miRNA qPCR is shown. The two-tailed unpaired student’s *t* test with Welch’s correction was used for the statistical analysis. The presented data are representative of two experiments. **E** Expression of *Pde2a* in MA9 cells and WT HSPCs in fragments per kilobase and per million mapped fragments (FPKM) as determined by RNA-seq is shown. The Wald’s test with Benjamin–Hochberg correction was used for the statistical analysis. All graphs show mean ± SEM.

We generated a murine MLL-AF9 model by transduction of bone marrow (BM) HSPCs with MLL-AF9-expressing retrovirus. MLL-AF9 leukemia cells were positive for c-Kit, GR-1, CD16/CD32, and CD11b and negative for CD3 expression, suggesting a myeloid progenitor origin of the leukemia cells (Fig. 1A). As expected from previous studies [27], DOT1L-inhibitors diminished cell survival of MLL-AF9 cells (Supplementary Fig. 1A). To validate our model, we performed proteomics and observed differential protein expression in MLL-AF9 cells (Supplementary Fig. 1B, Supplementary Table 2). The most upregulated protein was 15-hydroxyprostaglandin dehydrogenase (HPGD), suggesting a role for the HPGD pathway in MLL-AF9 leukemogenesis (Supplementary Fig. 1B). We performed Ingenuity Pathway Analysis of differentially expressed proteins and found that pathways involved in Cell Death and Survival (*P* < 2.74e^−3^), Hematological System Development and Function (*P* < 2.60e^−3^), and Hematological Diseases (*P* < 2.60e^−3^) were deregulated (Supplementary Fig. 1C). Transcriptomics and Gene Set Enrichment Analyses showed enrichment of MLL-AF9 targets [28] in the upregulated fraction of genes in MLL-AF9 cells compared to WT HSPCs (Supplementary Fig. 1D). In contrast, myeloid differentiation factors such as *C/ebpa*, *Csf1r*, *Csf3r,* and *Id2* were downregulated (Supplementary Fig. 1E). In concordance with previous studies [22, 29], we found enrichment of PRC1 and PRC2 targets, class-I (EZH2-controlled) and class-II (EZH2-independent) PcG target genes in the set of downregulated genes in MLL-AF9 cells compared to WT HSPCs (Supplementary Fig. 1F).

*MIR139* is downregulated in AML patients including cases with MLL rearrangements and in THP-1 cells that express MLL-AF9 compared with normal HSPCs and myeloid cells including monocytes, macrophages, and neutrophils [13]. We therefore asked whether genomic *Mir139* deletion collaborates with MLL-AF9 in AML outgrowth. *Mir139*KO mice developed normally and had expected HSPC counts and hematopoietic cell types in peripheral blood and BM [25]. Next, we asked whether *Mir139* influences genes that have potential effects on pathways other than those that are critical for hematopoiesis. We found that WT and *Mir139*KO HSPCs were indistinguishable at the transcriptome and proteome level, including *Pde2a* host gene expression (Fig. 1B, Supplementary Fig. 2A, Supplementary Table 3). These data suggest that *MIR139* is expressed at too low levels in normal HSPCs to sufficiently downregulate its targets to detectable levels. In contrast, genomic deletion of *Mir139* enhanced primary and secondary colony-forming capacity (more colonies) and proliferation (larger colonies) of MLL-AF9 cells (Fig. 1C). Strikingly, we observed a >60% reduction of *miR-139-3p* and *miR-139-5p* expression in MLL-AF9 cells, suggesting that MLL-AF9 silences *Mir139* (Fig. 1D). *Pde2a* expression in MLL-AF9 was unchanged, indicating that *Mir139* and the host gene are not co-regulated (Fig. 1E). Together, these results indicate that *Mir139* is a target of MLL-AF9.

### *MiR-139* upregulation and repression of *miR-139* targets eliminate MLL-AF9 AML

**Fig. 2:**
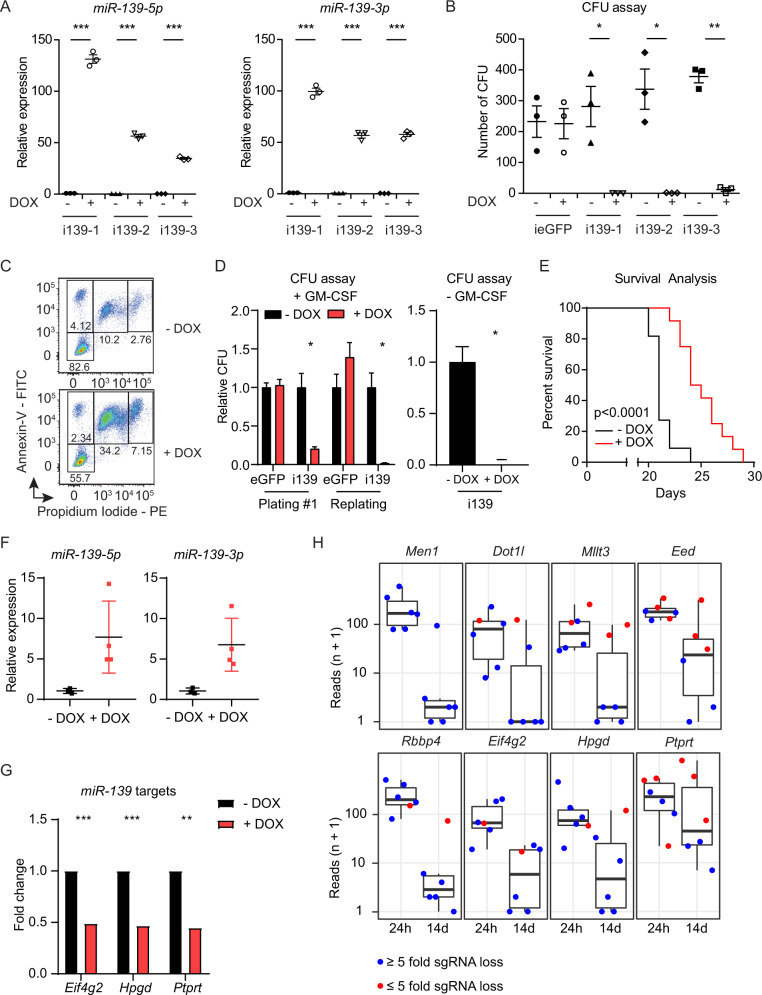
*MiR-139* upregulation and repression of *miR-139* targets eliminates MLL-AF9 AML in vitro and inhibits leukemogenesis in mice. **A** Expression levels of *miR-139-5p* and *miR-139-3p* in MLL-AF9-i139 clones (*n* = 3), treated with DOX (5 µg/mL, +) relative to snRNA U6 and mock-treated (−) cells, as determined by miRNA qPCR in triplicate, are shown. The two-tailed unpaired student’s *t* test was used for the statistical analysis. **B** The number of colony-forming units (CFU) of MLL-AF9-ieGFP and MLL-AF9-i139 (*n* = 3) HSPC clones treated with doxycycline (DOX; 5 µg/mL, (+) or mock-treated (−) as control per 8000 cells plated are shown. Data represent the average of three independent experiments. The two-tailed paired student’s *t* test was used for the statistical analysis. **C** Representative flow cytometry plot of MLL-AF9-i139 cells with or without DOX stained with propidium iodide and Annexin-V. Data are representative of three independent clones. **D** Relative number of colony-forming units (CFU) of MLL-AF9-ieGFP and MLL-AF9-i139-1 cells in triplicate, treated with Doxycycline (DOX; 5 µg/mL, (+)) or mock-treated (−) as control per 8000 cells plated are shown of the first plating and the subsequent replating. The cells were cultured with IL-3, IL-6, GM-CSF, and SCF (left panel) or with IL-3, IL-6, and SCF (right panel). The two-tailed paired student’s *t* test was used for the statistical analysis. **E** Kaplan–Meier plot showing the survival of mice, transplanted with MLL-AF9-GFP-TetR-KRAB-i139 LSK cells and treated with DOX (4 mg/kg) (red, n = 11) or mock-treated (black, *n* = 11). The Mantel–Cox test was used for the statistical analysis. **F** Expression levels of *miR-139-5p* and *miR-139-3p* in leukemia cells from mice treated with DOX (*n* = 4) in **E** relative to mock-treated and snRNA U6 are shown. Tissues were analyzed from mice when moribund. **G** Fold change of *Eif4g2* (Padj: 0.0002)*, Hpgd* (Padj: 0.0006), and *Ptprt* (Padj: 0.005) expression in DOX-treated (+DOX) MLL-AF9-i139 clones (*n* = 3) relative to mock-treated (−DOX) clones (*n* = 3) as determined by RNA-seq is shown. The Wald’s test with Benjamini–Hochberg correction was used for the statistical analysis. **H** Boxplots showing the reads (*n* + 1) of the total six sgRNAs targeting *Men1*, *Dot1l*, *Mllt3*, *Eed*, *Rbbp4*, *Eif4g2*, *Hpgd,* and *Ptprt* in MLL-AF9 cells 24 h or 14 days post transduction. Per gene, the order of the sgRNAs in the 24 h sample corresponds to the same sgRNAs in the 14d sample. Blue circles indicate a loss of >5 fold, while red circles indicate a loss of <5 fold of the sgRNA. All graphs depict mean ± SEM.

To determine effects of *miR-139* expression on MLL-AF9 cell survival, we generated (DOX)-inducible *miR-139-*expressing MLL-AF9 clones (MLL-AF9-i139). DOX-treatment of MLL-AF9-i139 cells induced *miR-139-5p* (up to 130 Fold) and *miR-139-3p* (up to 100 Fold) expression (Fig. 2A). DOX-treated MLL-AF9-i139 cells lost their colony-forming capacity, whereas DOX-inducible eGFP expressing MLL-AF9 control cells (MLL-AF9-ieGFP) expanded as expected (Fig. 2B). DOX-mediated induction of *miR-139* expression caused cell death and apoptosis of MLL-AF9-i139 cells as indicated by Annexin-V positivity and loss of replating capacity (Fig. 2C, D). In addition, induction of *miR-139* expression in MLL-AF9-i139 cells cultured in expansion medium without GM-CSF, stimulating mainly leukemia stem cell (LSC) expansion, completely blocked colony outgrowth (Fig. 2D). Similarly, MLL-AF9-i139 cells generated from hematopoietic stem cells (Lin^−^Sca-1^+^c-Kit^+^, LSK)(LSK-MLL-AF9-i139) showed loss of cells upon *miR-139* induction (Supplementary Fig. 2B). Together, our results suggest that *miR-139* expression also eliminates LSCs.

Next, we asked whether *miR-139* expression inhibits MLL-AF9 leukemia development in mice. Mice transplanted with MLL-AF9-i139 cells developed AML with a short latency of 3 weeks. However, DOX-mediated *miR-139* induction significantly delayed leukemia development of transplanted mice (Fig. 2E). Mice had malignant MLL-AF9-i139 cells in BM (Supplementary Fig. 2C), blood, and spleen (data not shown). Leukemia cells from mice that were treated with DOX showed an average *miR-139-5p* and *miR-139-3p* overexpression induction of eightfold and sevenfold, respectively (Fig. 2F), which suggests that this level of upregulation in escaping MLL-AF9-i139 cells is not sufficient to eliminate MLL-AF9 cells in mice. In full agreement, secondary transplantation experiments with these AML cells show no difference in latency between DOX-treated and non-treated controls (data not shown). These data indicate the tumor-suppressing activities of *miR-139* in MLL-AF9 AML.

To gain mechanistic insight, we performed mRNA sequencing of DOX-treated MLL-AF9-i139 and untreated clones and found that a subset of genes were differentially expressed between these populations (Supplementary Fig. 2D, Supplementary Table 4). We established that genes that inhibit cell cycle progressions, such as *Btg2, Cdkn1a,* and *Cdkn2d*, and pro-apoptotic genes such as *Bmf*, *Dusp1*, *Rnasel*, *Trp53inp1*, *Ypel3,* and *Zc3h12a* were upregulated in DOX-treated MLL-AF9-i139 cells (Supplementary Table 4, Supplementary Fig. 2F). Furthermore, we noted that predicted *miR-139* targets such as *Eif4g2*, *Ptprt* and *Hpgd* were downregulated in *miR-139* expressing MLL-AF9 cells (Fig. 2G, Supplementary Table 4).

Next we wanted to validate *miR-139* targets that mediate its tumor suppressor activity in MLL-AF9 cells. We performed a genome-wide CRISPR-KO screen to identify genes critical for MLL-AF9 survival or proliferation (Supplementary Fig. 2E). After 14 days, cells with sgRNAs that target such genes will be lost. Confirming the effectivity of our sgRNA screen, we found that sgRNAs targeting *Men1* (six sgRNAs), a crucial co-factor for MLL-AF9 [30], the PRC2 subunits *Eed* (three out of six sgRNAs) and *Rbbp4* (five out of six sgRNAs), *Dot1l* (five out of six sgRNAs) and *Mllt3* (four out of six sgRNAs) were lost (Fig. 2H, Supplementary Table 5). We found that MLL-AF9 cells with sgRNAs targeting *Ptprt* (three out of six) *Hpgd* (five out of six) and *Eif4g2* (five out of six) were also lost, suggesting that expression of our identified *miR-139*-targets is essential for cell survival (Fig. 2H, Supplementary Table 5). Validation experiments with single sgRNAs targeting *Hpgd*, *Eif4g2,* and *Ptprt* in colony-forming unit assays confirmed these results and showed that the depletion of these *miR-139* target genes could, at least in part, phenocopy *miR-139* expression in MLL-AF9 cells (Supplementary Fig. 2G). Furthermore, luciferase-based miRNA target validation assays confirmed that *Hpgd* and, *Eif4g2* and *Ptprt* are direct targets of *miR-139* (Supplementary Fig. 2H). Taken together, these data show that *miR-139* expression and the downregulation of *miR-139* targets inhibit MLL-AF9 leukemia.

### *Mir139* is epigenetically silenced by PRC2 in MLL-AF9 cells

We next investigated how *MIR139* expression is regulated. *MIR139* is epigenetically silenced in AML, including the THP-1 cell line that expresses MLL-AF9 [13]. We hypothesized that *Mir139* silencing by histone modifications is critical for MLL-AF9 AML. To test this, we treated MLL-AF9 and MLL-AF9-*Mir139*KO cells with Trichostatin-A (TSA) or valporic acid (VPA), which are inhibitors of class-I and II histone deacetylases. We found that MLL-AF9 cells treated with either TSA or VPA, were significantly reduced in their colony-forming capacity compared with MLL-AF9-*Mir139*KO cells (Supplementary Fig. 3A). These results suggest a role for *MIR139* epigenetic silencing in MLL-AF9 AML expansion and survival.

**Fig. 3:**
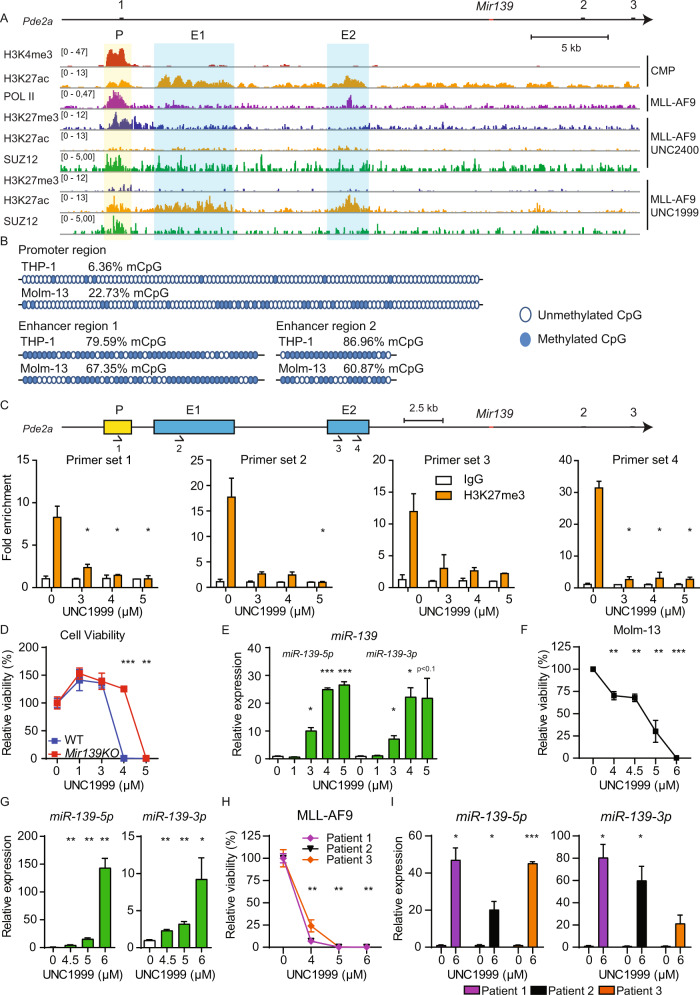
*Mir139* is epigenetically silenced by PRC2 in MLL-AF9 cells. **A** ChIP-seq reads of the *Pde2a* locus (chr7:108,596,060–108,645,660) are depicted. The top two tracks show H3K4me3 (red) and H3K27Ac (orange) enrichment in common myeloid progenitors (CMPs). The next track indicates RNA Polymerase II (POL-II) binding (purple) of MLL-AF9 cells. The next three tracks depict H3K27me3 (blue), H3K27Ac and SUZ12 (green) enrichment in MLL-AF9 cells treated with UNC2400 (inactive PRC2 inhibitor). The bottom three tracks show the same data of MLL-AF9 cells treated with UNC1999 (active PRC2 inhibitor). Scale bar indicates 5 kilobases (kb). Black bars indicate exons. **B** The methylation status of CpGs in the *PDE2A* promoter region, enhancer region 1 (E1) and E2 of THP-1 and Molm-13 cells is depicted. Closed blue circles indicate methylated CpG, open blue circles indicate unmethylated CpG. **C** Schematic overview of the *Pde2a* locus (chr7:108,596,060–108,645,660). Fold enrichment of H3K27me3 (orange) and control IgG (white) antibody binding to the indicated regions in MLL-AF9 cells treated with depicted concentrations of UNC1999 as determined by ChIP-qPCR is shown. Arrows indicate the primer sets. The scale bar indicates 2.5 kb. **D** Viability of WT or *Mir139*KO MLL-AF9 cells treated with UNC1999 relative to mock-treated WT or *Mir139*KO MLL-AF9 cells is shown. Data are representative of three experiments. **E** Expression levels of *miR-139-3p* and *miR-139-5p* in MLL-AF9 cells treated with UNC1999 relative to snRNA U6 and untreated MLL-AF9 cells are shown. Data are representative of three experiments. **F** Viability of Molm-13 cells treated with UNC1999 relative to mock-treated Molm-13 cells, is shown. Depicted data are representative of three experiments. **G** Expression levels of *miR-139-3p* and *miR-139-5p* in Molm-13 cells treated with UNC1999 relative to snRNA U6 and untreated Molm-13 cells are shown. Depicted data are representative of three experiments. **H** Viability of UNC1999-treated primary MLL-AF9 patient samples (*n* = 3) relative to untreated cells is shown. **I** Expression levels of *miR-139-5p* and *miR-139-3p* in primary MLL-AF9 patient samples (*n* = 3) treated with UNC1999 relative to snRNA U6 and untreated cells are shown. All graphs show mean ± SEM. The two-tailed unpaired student’s *t* test was used in **C**–**E** and **G**. The two-tailed paired student’s *t* test was used in **F**, **H** and **I**.

Next, we investigated the epigenetic landscape of *Mir139* by reanalyzing publicly available ChIP-seq data sets from mouse MLL-AF9 cells and WT HSPCs [29, 31, 32]. *Mir139* is located in intron-1 of the *Pde2a* host gene with the same orientation, downstream of the highly conserved putative *Pde2a* promoter (P). As expected, H3K4me3 peaks overlapped with P and H3K27Ac peaks overlapped with enhancer region-1 (E1) and E2 (Fig. 3A). In these genomic regions we noted two POL-II peaks: one located at the first exon of *Pde2a* and one on E2, in intron-1 of *Pde2a* (Fig. 3A). We found H3K27me3 levels, which are associated with epigenetic silencing, at P, E1, and E2 in MLL-AF9 cells (Fig. 3A). MLL-AF9 cells treated with UNC1999, a specific inhibitor of the methyltransferases EZH1 and EZH2 [29], showed decreased H3K27me3 and increased H3K27Ac levels compared to UNC2400 control inhibitor-treated cells. In addition, ChIP-seq data revealed SUZ12 binding to P, E1, and E2, which was decreased in cells treated with UNC1999 (Fig. 3A).

Aberrant enhancer DNA methylation is acquired during malignant transformation [33]. PRC2, a chromatin-modifying enzyme that catalyzes histone-H3 lysine 27 trimethylation, is known to have a critical role in MLL-AF9 pathogenesis [22, 23] and prefers binding to methylated DNA via AEBP2 [34]. To address the role of PRC2 in context of *MIR139*, we determined the CpG methylation status of human MLL-AF9 cell lines Molm-13 and THP-1. We observed that a small percentage of CpGs present in the *MIR139* promoter region of Molm-13 (22.73%) and THP-1 (6.36%) are methylated (Fig. 3B). Although there is a lower density of CpGs in the enhancer regions close to *MIR139*, we detected an increased percentage of methylated CpGs in E1 (67% and 79%) and E2 (60% and 87%) in Molm-13 cells and THP-1 cells, respectively (Fig. 3B).

To experimentally validate the relevance of PRC2-mediated silencing of *Mir139*, we treated MLL-AF9 cells with UNC1999 and determined cell survival compared with MLL-AF9-*Mir139*KO cells. UNC1999 activity was confirmed by reduced H3K27me3 levels at the P, E1, and E2 (Fig. 3C). We observed a decreased viability of MLL-AF9 cells compared with MLL-AF9-*Mir139*KO cells, strongly suggesting that UNC1999 treatment activates *miR-139* expression (Fig. 3D). Indeed, loss of cell viability coincided with up to 25-fold increased levels of *miR-139-5p* and up to 20-fold increased *miR-139-3p* expression (Fig. 3E). Notably, treatment of MLL-AF9 cells with UNC2400 did not upregulate *miR-139* and sustained cell viability (Supplementary Fig. 3B, C). Expression of *miR-139-5p* was not induced when WT HSPCs were treated with UNC1999 (Supplementary Fig. 3D). *Cdkn2a* is a known target of PRC2 in MLL-AF9 AML [22, 23]. In agreement, we observed a three to sixfold upregulation of *Cdkn2a* (Supplementary Fig. 3E). The expression of *Pde2a* was unaffected by UNC1999 (Supplementary Fig. 3F). This mechanism is conserved between mouse and human, because comparable results were obtained with Molm-13 (Fig. 3F, G), THP-1 (Supplementary Fig. 3G, H) and primary MLL-AF9 patient samples (Fig. 3H, I). In addition, UNC1999 treatment of a panel of AML cell lines caused decreased cell viability and increased *miR-139* expression (Supplementary Fig. 3I, J). This suggests that PRC2-mediated *MIR139* silencing may not be exclusive for MLL-AF9 AML. Taken together, these data indicate that MLL-AF9 silences *MIR139* by a PRC2-mediated mechanism.

### E1 and E2 control the expression of *Mir139*

**Fig. 4:**
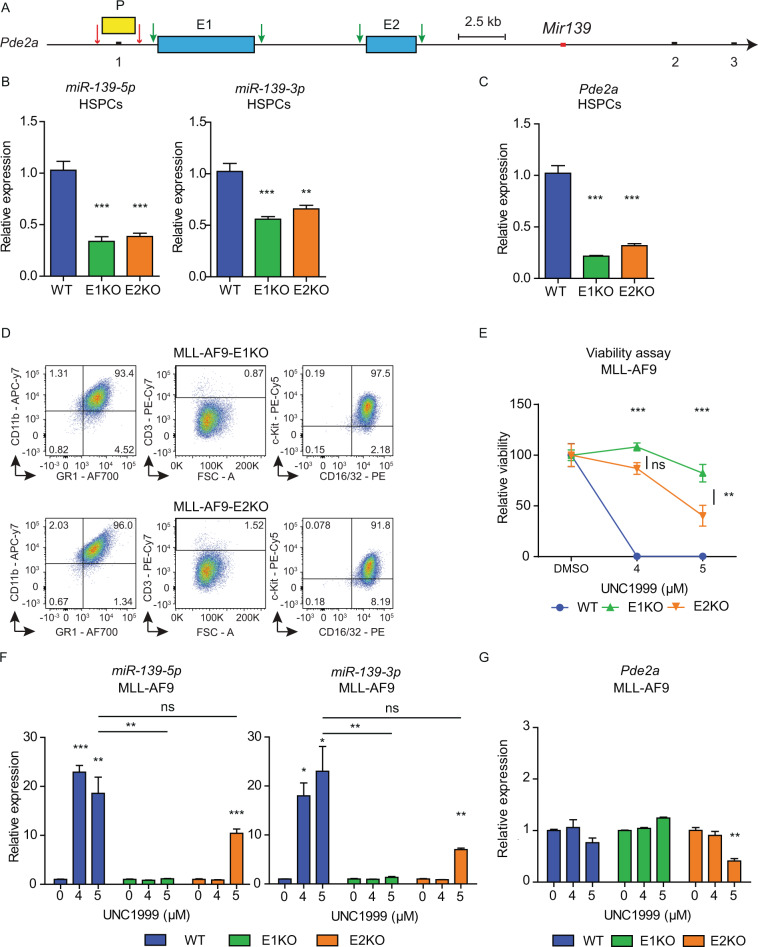
E1 and E2 are both critical for the transcriptional regulation of *Mir139*. **A** Schematic representation of CRISPR-Cas9 KO strategy to generate PKO, E1KO, and E2KO mice. Red arrows indicate sgRNAs targeting the promoter region and exon-1 of *Pde2a*. Green arrows indicate sgRNAs targeting E1 and E2. The first three exons of *Pde2a* (black bars) are indicated with numbers 1, 2, and 3. The *Mir139* encoding sequence is depicted in red. The scale bar indicates 2.5 kb. **B** Expression levels of *miR-139-3p* and *miR-139-5p* in Enhancer 1 KO (E1KO) and E2KO HSPCs relative to snRNA U6 and WT HSPCs are shown **C**. Expression of *Pde2a* relative to *Gapdh* and to WT HSPCs, in E1KO and E2KO HSPCs isolated from three mice per condition is shown. **D** Flow cytometric analysis of surface marker expression of MLL-AF9-E1KO and MLL-AF9-E2KO cells stained for myeloid lineage markers CD11b, CD16/32, c-Kit, GR-1, and lymphoid lineage marker CD3. **E** Viability of MLL-AF9 WT, E1KO, and E2KO MLL-AF9 cells treated with indicated concentrations of UNC1999 relative to the mock-treated condition cells is shown. The ANOVA analysis with Bonferroni correction was used for statistical analysis. *In top of graph: comparisons of E1KO and E2KO with WT. Presented data are representative of three independent experiments. **F** Expression levels of *miR-139-3p* and *miR-139-5p* in WT, E1KO, and E2KO MLL-AF9 cells treated with indicated concentrations of UNC1999 relative to snRNA U6 and mock-treated cells are shown. Depicted data are representative of three experiments. **G** Expression of *Pde2a* in WT, E1KO, and E2KO MLL-AF9 cells, treated with indicated concentrations of UNC1999 relative to *Gapdh* and mock-treated cells is shown. Depicted data are representative of three experiments. The two-tailed unpaired student’s *t* test with Welch’s correction was used for statistical analysis in **B**–**F** and **G**. All graphs indicate mean ± SEM.

To further investigate the role of the P, E1, and E2 in *Mir139* expression, we deleted these genomic regions in mice (Fig. 4A). We were unable to generate mice with genomic deletion of the *Pde2a* promoter, which overlaps with exon-1, suggesting that a *Pde2a* KO is embryonically lethal. In contrast, E1 and E2 deletion mutant mice were viable and developed normally. Under physiological conditions, deletion of either E1 or E2 caused a >60% reduction of *miR-139-5p* expression, a 40–50% reduction of *miR-139-3p* expression in WT HSPCs and a 70–80% reduction of *Pde2a* expression (Fig. 4B, C). Next, we investigated whether E1 and E2 are involved in *Mir139* silencing in MLL-AF9 cells. Therefore, we generated MLL-AF9-E1KO and MLL-AF9-E2KO cells. Similar to MLL-AF9 cells, these cells were positive for GR-1, CD11b, CD16/32, c-Kit, and negative for CD3 (Fig. 4D). Next, we treated these cells with UNC1999 and noted reduced cell death of MLL-AF9-E1KO and MLL-AF9-E2KO cells compared to MLL-AF9 cells, suggesting that the *miR-139* induction is affected (Fig. 4E). Indeed, we found that upon UNC1999 treatment, *miR-139-5p* and *miR-139-3p* were not upregulated in MLL-AF9-E1KO cells (Fig. 4F). Notably, *Pde2a* expression was not affected in UNC1999-treated MLL-AF9-E1KO cells (Fig. 4G). MLL-AF9-E2KO cells showed a significantly reduced *miR-139-5p* and *miR-139-3p* induction, which coincided with slightly reduced *Pde2a* levels with 5 µM UNC1999 (Fig. 4F, G). Together, these data indicate that both E1 and E2 regions control *Mir139* expression in AML.

### POLR2M silences *MIR139* in MLL-AF9 cells

**Fig. 5:**
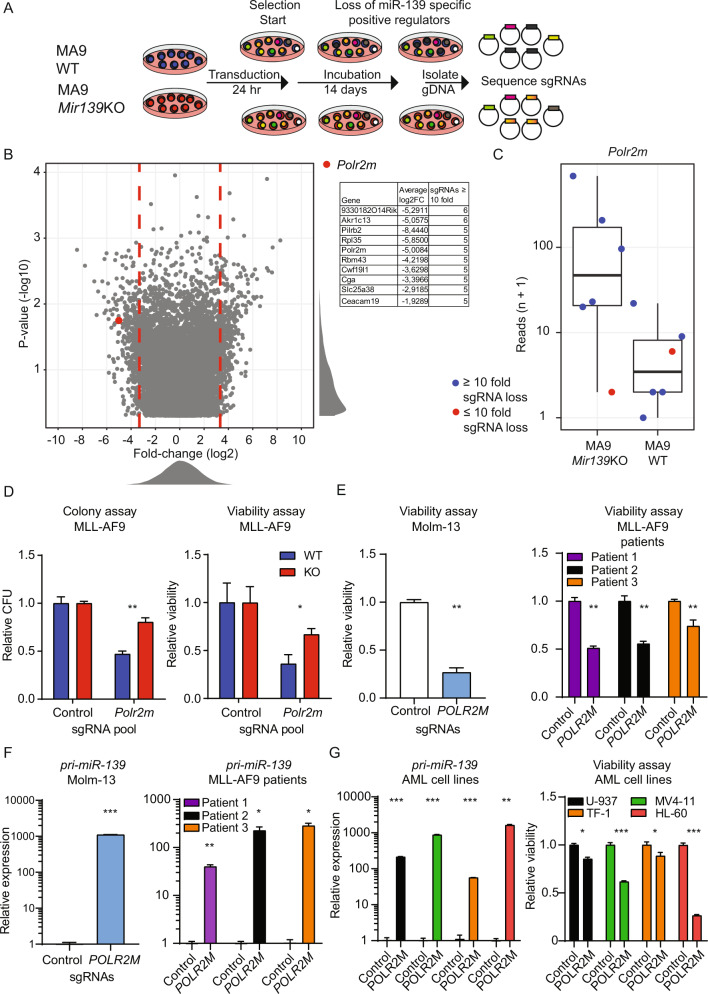
POLR2M silences *Mir139* in MLL-AF9 cells. **A** Schematic overview of the sgRNA library screen with MLL-AF9 WT (MA9 WT) and MLL-AF9 *Mir139*KO (MA9 *Mir139*KO) cells. **B** Volcano plot showing –log10 *P* value (*Y* axis) and average log2 fold change (*X* axis) of all targeted genes in MLL-AF9 WT relative to MLL-AF9-*Mir139*KO 14 days post transduction. The red closed circle indicates *Polr2m*. Red dashed lines indicate a fold change of ±10. Top 10 of enriched genes are shown. **C** Boxplots showing the reads (*n* + 1) of six sgRNAs targeting *Polr2m* in MA9 WT and MA9 *Mir139*KO cells. The order of the sgRNAs in the MA9 *Mir139*KO sample corresponds to the same sgRNAs in the MA9 WT sample. Blue circles indicate a loss of ≥10 fold per sgRNA, while red circles indicate a loss of ≤10 fold of the sgRNA. **D** MLL-AF9 cells and MLL-AF9 *Mir139*KO cells were transduced with three viruses expressing sgRNAs targeting *Polr2m* or three viruses expressing control sgRNAs. The left panel shows the colony-forming units (CFU) of *Polr2m*-targeted MLL-AF9 cells relative to MLL-AF9 *Mir139*KO cells. The right panel shows the viability of *Polr2m*-targeted MLL-AF9 cells relative to MLL-AF9 *Mir139*KO cells (*Y* axis). The two-tailed unpaired student’s *t* test with Welch’s correction was used for statistical analysis. The graphs are representative of three independent experiments. **E** Viability of *POLR2M*-targeted Molm-13 cells (left panel) or primary MLL-AF9 patient samples (right panel) relative to control targeted cells. The two-tailed paired student’s *t* test was used for the statistical analysis. Data are representative of two/three experiments. **F** Expression levels of pri-*miR-139* in *POLR2M*-targeted Molm-13 cells (left panel) or primary MLL-AF9 patient samples (right panel) relative to control targeted cells. The two-tailed unpaired student’s *t* test with Welch’s correction was used for statistical analysis. The graphs are representative of two/three independent experiments. **G** Expression levels of pri-*miR-139* in *POLR2M*-targeted U937, MV4-11, TF-1, or HL-60 cells relative to control targeted cells (left panel). Viability of *POLR2M*-targeted U937, MV4-11, TF-1, or HL-60 cells relative to control targeted cells (right panel). The two-tailed unpaired student’s *t* test with Welch’s correction was used for statistical analysis. The graphs are representative of two independent experiments. All graphs are depicted as mean ± SEM.

To identify novel factors that are involved in transcriptional silencing of *Mir139* in MLL-AF9 cells, we performed a genome-wide CRISPR-KO screen [35]. We used MLL-AF9-*Mir139*KO cells as a reference, which allowed for screening of factors that specifically silence *Mir139* transcription. We expected that inactivation of genes encoding for factors responsible for *Mir139* silencing would upregulate *miR-139* expression and induce apoptosis in MLL-AF9 cells, but not in MLL-AF9-*Mir139*KO cells (Fig. 5A). We selected all sgRNAs with 10 or more reads in MLL-AF9 cells and MLL-AF9-*Mir139*KO cells (MLL-AF9-WT + MLL-AF9-*Mir139*KO ≥ 10) after 14 days of incubation (Fig. 5B). We reasoned that a higher number of sgRNAs lost per gene will increase the chance that these genes are relevant for *Mir139* regulation. According to our analysis of control sgRNAs, the chance that a loss of five sgRNAs or more per gene is an artifact is >0.0016%. Out of the genes shown in Fig. 5B, we identified only 16 genes targeted by 5–6 sgRNAs that were significantly reduced by ≥ 10 fold in MLL-AF9 cells compared with MLL-AF9-*Mir139*KO cells (Supplementary Fig. 4A, Supplementary Table 6). Among these 16 genes, RNA Polymerase 2 Subunit M (*Polr2m*), involved in stable transcriptional pausing of POL-II [36–38], was the only negative transcriptional regulator and was selected for further validation (Fig. 5C). CRISPR-Cas9-mediated KO of *Polr2m* reduced colony outgrowth and cell viability of MLL-AF9 cells compared with MLL-AF9-139-KO cells (Fig. 5D). In agreement, POLR2M depletion reduced the viability of Molm-13 cells and primary MLL-AF9 cells from patients (Fig. 5E). Furthermore, sgRNA-mediated targeting of *POLR2M* caused an increase of *pri-miR-139* and mature *miR-139* expression in Molm-13 cells and in primary MLL-AF9 patient samples compared with control sgRNA treated cells (Fig. 5F, Supplementary Fig. 4B, C). In these experiments, *Pde2a* expression was not affected by *Polr2m* depletion (Supplementary Fig. 4D). These results were confirmed in various human AML cell lines with different oncogenic aberrations (Fig. 5G, Supplementary Fig. 4D). Collectively, these data suggest that POLR2M is a silencing factor of *MIR139* downstream of PRC2, an oncogenic mechanism that may not be exclusive for MLL-AF9 AML.

### POLR2M binds to E1, E2, and the transcriptional start site of *MIR139*

**Fig. 6:**
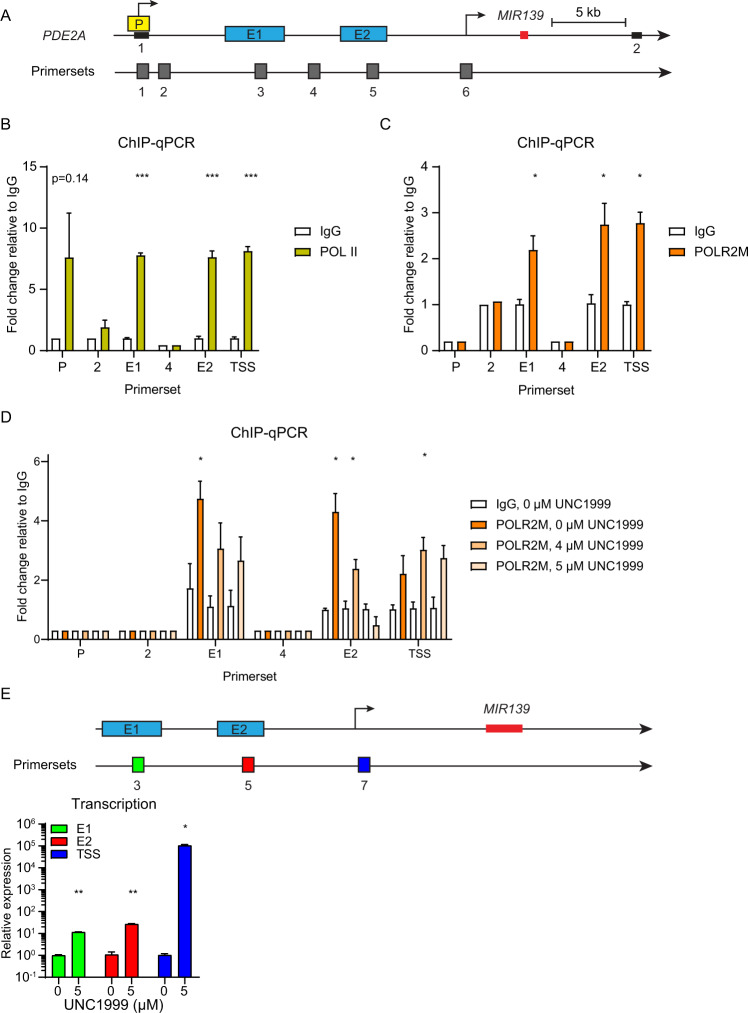
POLR2M binds to E1, E2, and the TSS. **A** Schematic representation of the *PDE2A* locus (chr11:72,605,000–72,644,500) with the indicated promoter (P, yellow) and enhancer (E1 or E2, blue) regions and transcriptional start sites (arrows). The first two exons of *PDE2A* are depicted with a black horizontal bar. The *MIR139* encoding sequence is depicted in red. The location of the primer sets with the respective numbers used for the experiments in this figure is depicted in gray. **B**, **C** Fold enrichment of POL-II (yellow) and POLR2M (orange) on the depicted loci relative to IgG (white) as determined by ChIP-qPCR in Molm-13 cells are shown. Data are representative of three experiments. **D** Same as **B** and **C**, except that Molm-13 cells were treated with indicated concentrations of UNC1999 (different shades of orange). Data are representative of two independent experiments. **E** Schematic overview of the *MIR139* locus. The primer sets to detect transcripts coming from E1 (green), E2 (red), and TSS (blue) are shown. The relative expression of transcripts in UNC1999-treated Molm-13-treated cells is shown below. The two-tailed unpaired student’s *t* test with Welch’s correction was used for statistical analysis in **B**–**E**. Data are representative of three experiments. All graphs are depicted as mean ± SEM.

**Fig. 7:**
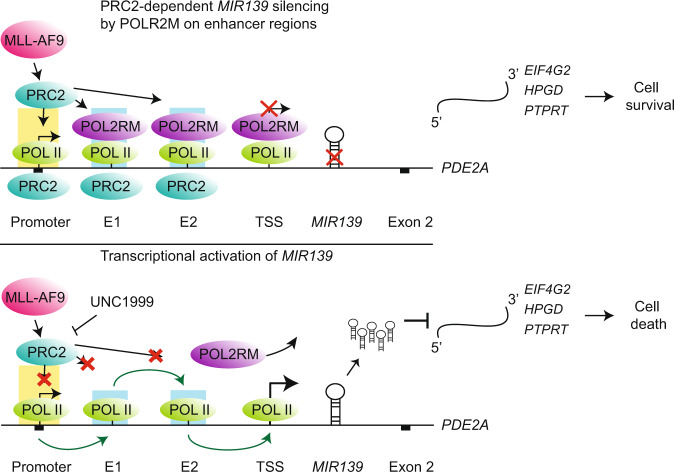
Schematic illustration of the model. In the top panel, the *MIR139* silencing mechanism in MLL-AF9 AML is shown. MLL-AF9 recruits PRC2 to the promoter, E1, E2, and the TSS of the *PDE2A* locus. POLR2M is recruited to E1, E2, and the TSS. POLR2M silences the transcription of *MIR139*, while the transcription of the host gene is unaffected. In the bottom panel, the mechanism of transcriptional activation by PRC2 inhibition and POLR2M depletion is shown. POLR2M depletion and PRC2 inhibition by UNC1999, which abrogates POLR2M interaction with the enhancers, results in transcription of *MIR139*. Increased *miR-139* levels results in downregulation of *EIF4G2*, *PTPRT,* and *HPGD* and lead to cell death of MLL-AF9 cells.

Because POLR2M binds to POL-II to mediate stable transcriptional pausing [36–38], we hypothesized that POLR2M is bound at the POL-II interaction sites within the *MIR139* locus. We used publicly available data sets to determine enrichment of H3K4me3, H3K27Ac, H3K4me1, and POL-II binding on E1, E2, and P regions (Supplementary Fig. 5). In our analysis, we included a previously identified *MIR139* transcriptional start site (TSS) [15] downstream of E2 (Fig. 6A). ChIP-qPCR experiments with chromatin isolated from Molm-13 cells showed significant enrichment of POL-II at P, E1, and E2 regions and the TSS downstream of E2 (Fig. 6B). We observed POLR2M interactions with E1, E2, and the TSS downstream of E2, whereas this was not observed at P (Fig. 6C). Treatment of Molm-13 cells with UNC1999 abrogated POLR2M binding to E1 and E2, but not to the TSS downstream of E2 (Fig. 6D). In UNC1999-treated Molm-13 cells we found strong induction of transcripts originating from the TSS, which was minor at E1 or E2 (Fig. 6E). These data suggest that E1 and E2 in UNC1999-treated cells, strongly induces the transcription by direct interaction with the downstream TSS of *MIR139* (Supplementary Fig. 5B). Taken together, these data indicate that POLR2M-binding to E1 and E2 silences the transcription of *MIR139* at the TSS downstream of PRC2 in MLL-AF9 AML, which is also illustrated in our model (Fig. 7).

## Discussion

Our study has revealed key aspects of the molecular regulation of the tumor suppressor *MIR139* in MLL-AF9 leukemia. We describe that expression of *miR-139* in MLL-AF9 cells caused apoptosis, abolished colony formation in vitro, and inhibited MLL-AF9 leukemia in vivo. Besides the previously published target *Eif4g2* [13], we identified *Hpgd* and *Ptprt* as novel *miR-139*-controlled targets of which depletion in MLL-AF9 AML at least in part phenocopied *miR-139* expression. Our data suggest a mechanism of *MIR139* silencing in MLL-AF9 AML in which PRC2 activity and recruitment of POLR2M at the E1, E2 inhibit *MIR139* expression to promote oncogenesis (Fig. 7).

There is emerging evidence that *MIR139* is a tumor suppressor in various types of solid cancer [15, 39] and leukemia [12–14]. *Eif4g2* is a target of *miR-139-5p* in AML [13], which is confirmed in our study. The RNA-binding protein *Elavl1* is commonly found as a target for *miR-139-3p* in solid cancer [39] and upon ICL-induced stress in HSPCs [12]. In the present study however, *Elavl1* is not differentially expressed between HSPCs and MLL-AF9 cells and when *miR-139* expression is induced in MLL-AF9-139 cells. This suggests that *miR-139* dependent target regulation is cell type- and context-dependent, which has been previously observed for other miRNAs and cell types [40–42].

PRC2-mediated repression of tumor suppressor genes, including *Cdkn2a* (p16^INK4A^), *Cdkn2b* (p15^INK4B^), *Gata2* and *Egr1*, is a critical event in MLL-AF9 AML [22, 23, 43]. We provide evidence that *Mir139* is a tumor suppressor that is targeted by PRC2 downstream of MLL-AF9. First, MLL-AF9 expression in HSPCs caused strong repression of *Mir139* via PRC2. This is a critical step in oncogenic transformation as we demonstrated that *miR-139* induction in MLL-AF9 cells caused apoptosis, loss of colony-forming capacity and inhibition of leukemogenesis in mice. We found that escaping AML cells in the DOX-treated mice have a *miR-139* upregulation of only less than eightfold due to unknown reasons, in contrast to the 50-100-fold upregulation as found in vitro experiments. Apparently, an eightfold induction of *miR-139* is not sufficient to cause apoptosis in the transplanted AML cells, allowing this aberrant sub-clone to give rise to full-blown leukemia. Second, genetic *Mir139* deletion partly rescued the oncolytic effect of PRC2 inhibition in MLL-AF9 cells. Notably, the enhanced expansion capacity of MLL-AF9-*Mir139*KO cells compared with MLL-AF9 cells indicate that complete loss of *Mir139* is a selective advantage for leukemia outgrowth in vitro. These results explain clinical data showing that AML patients with the lowest *miR-139* expression levels have a poor prognosis [13]. Third, inhibition of methyltransferases EZH1 and EZH2 by UNC1999 greatly induced *miR-139* expression in MLL-AF9 AML patient samples and other subtypes of AML, demonstrating direct silencing of *Mir139* by PRC2 in AML, which is in agreement with previous findings in solid cancers [44, 45]. This suggests that the PRC2-mediated *MIR139* silencing is a general mechanism in cancer and may be a key event in oncogenesis. Fourth, we identified *Eif4g2*, *Ptprt*, and *Hpgd* as *miR-139* targets, which are involved in MLL-AF9 cell survival. Deregulation of these three genes by mutation, disruption of miRNA-mediated regulation or genomic aberrations are frequently found in different types of cancer [13, 46–50]. Increased expression of *Eif4g2*, caused by *MIR139* silencing, enhances protein synthesis [46], which is essential for the proliferation of AML cells [13]. *Ptprt* controls JAK/STAT signaling by dephosphorylation of STAT3, which is frequently dysregulated in human cancer, including AML [51, 52]. The expression and phosphorylation of STAT3 beta (STAT3β), which is an isoform of STAT3 has tumor-suppressive activities in MLL-AF9 AML [53]. Therefore, as a result of *MIR139* silencing, STAT3β phosphorylation is potentially decreased, thereby contributing to MLL-AF9 oncogenesis. HPGD is an enzyme that converts PGE2 to 15-keto-PGE2 and has been associated with epithelial-to-mesenchymal transition and poor survival of patients with breast cancer and lung cancer [46, 54]. Furthermore, our proteomics data demonstrate HPGD as the most upregulated protein in MLL-AF9 cells compared with WT HSPCs, suggesting that PGE2 conversion by HPGD contributes to MLL-AF9 oncogenesis. At last, we further demonstrate that *miR-139* expression in MLL-AF9-i139 cells induces loss of colony-forming capacity and apoptosis. Our gene expression data show that genes that inhibit cell cycle progression, such as *Btg2* [55]*, Cdkn1a* [56], and *Cdkn2d* [57], and genes that promote apoptosis such as *Bmf* [58], *Dusp1* [59], *Rnasel* [60], *Trp53inp1* [61], *Ypel3* [62], and *Zc3h12a* [63] are upregulated upon *miR-139* expression and may explain this phenotype. Taken together, we conclude that *MIR139* is silenced via PRC2 and that this silencing mechanism induces different pathways via *miR-139* targets that contribute to cell survival and MLL-AF9 leukemogenesis.

Our data indicate that *Mir139* silencing is independent of the transcriptional regulation of *Pde2a*. This may be explained by the high percentage of methylated CpGs within E1 and E2 in MLL-AF9 cells, whereas the upstream promoter region is relatively less methylated. PRC2 prefers binding to methylated DNA via AEBP2 [34]. Accordingly, CpGs in the genomic regions overlapping with the POL-II binding sites in E1 and E2 were methylated in Molm-13 and THP-1 cells. In addition, we found that both enhancers are essential for PRC2-mediated *Mir139* silencing in MLL-AF9 cells. Thus, CpG methylation at these enhancers may be involved in the selective repression of *Mir139*, leaving the *Pde2a* host gene expression intact.

We found that *Pde2a* expression and intron-1 splicing did not result in *miR-139* expression in MLL-AF9 cells. These data suggest that in MLL-AF9 cells *pri-miR-139* is degraded. We did not observe *miR-139* induction upon PRC2 inhibition in MLL-AF9-E1KO cells and a reduced *miR-139* induction in MLL-AF9-E2KO, suggesting that factors involved in *miR-139* stability and processing may be recruited to the enhancers. Enhancer regions may play critical roles in the transcriptional regulation of miRNAs in cancer [64]. A recent study showed that DROSHA and DGCR8 interact with super-enhancer regions and boost miRNA production [65]. In agreement with these studies, our data suggest a mechanism in which the RNA-processing machinery is directly coupled to enhancer-mediated transcription of *MIR139*. This interaction may occur under cellular stress conditions resulting in increased *miR-139* expression and cell death to prevent oncogenic transformation.

POL-II is the key factor responsible for the transcription of miRNAs [66]. Although stable paused transcription seems a logical downstream consequence of PcG-mediated gene silencing [67], the factors involved in the molecular mechanism of PRC2-mediated transcriptional pausing remained unknown. We found that POLR2M is recruited in a PRC2-dependent fashion to E1 and E2 to silence *MIR139* expression. POLR2M interacts with the POL-II complex to pause transcription [38, 68, 69]. Promoter-proximal pausing of POL-II at TSSs has been correlated with H3K27me3 and PcG-silenced genes [70–72]. Paused POL-II complexes have also been found at enhancer regions [73]. Together, our study indicates that POLR2M interacts with E1 and E2 and silences *MIR139* expression in MLL-AF9.

Our data demonstrate in different ways that POLR2M is a direct silencer of *MIR139* in AML. First, *POLR2M* depletion in MLL-AF9 patient samples and human AML cell lines induced the expression of *pri-miR-139*. Although the levels of the mature *miR-139-5p* and *miR-139-3p* are low compared with experiments with UNC1999-mediated repression of PRC2, this could be explained by the fact that the CRISPR-Cas9-mediated depletion of *POLR2M* by lentiviral transduction is an asynchronous process. This causes a heterogeneous induction of *MIR139* expression and subsequent cell death, thereby underestimating *miR-139* levels. Second, *POLR2M*-depleted MLL-AF9 cells were lost in our CRISPR-Cas9 screen and validation experiments, whereas *POLR2M*-depleted MLL-AF9-*Mir139*KO cells survived, indicating that POLR2M-controlled survival is specifically mediated by the silencing of *MIR139*. Third, we showed that POLR2M is bound to the E1 and E2 regions and TSS of *MIR139*. Depletion of *POLR2M* at these loci was observed as a result of UNC1999-mediated inhibition of PRC2 and coincides with induction of *miR-139* expression and cell death.

In conclusion, we have identified key molecular mechanisms that control tumor suppressor *MIR139* in MLL-AF9 leukemia and report functionally relevant targets of *miR-139-*mediated cell death of MLL-AF9 cells. Furthermore, our findings reveal a PRC2-dependent POLR2M-mediated silencing mechanism of the *MIR139* tumor suppressor in MLL-AF9 leukemia. The specific interaction of POLR2M with the POL-II complex is essential for transcriptional inhibition [74]. Therefore, POLR2M would be interesting for therapeutic targeting, for instance by molecularly interfering with the interaction with POL-II, thereby activating *MIR139* expression and eliminating AML. Together, our findings highlight the importance of POLR2M-mediated silencing of *MIR139* in MLL-AF9 AML.

## Supplementary information

Supplementary Information

Supplementary Figure 1

Supplementary Figure 2

Supplementary Figure 3

Supplementary Figure 4

Supplementary Figure 5

Supplementary Table 1

Supplementary Table 2

Supplementary Table 3

Supplementary Table 4

Supplementary Table 5

Supplementary Table 6

## Data Availability

The NGS data generated in this paper is available from the GEO database with the following identifiers. GSE160404: RNA-seq of WT HSPCs, *Mir139*KO HSPCs and MLL-AF9 cells GSE160405: RNA-seq of MLL-AF9-i139 cells treated with and without DOX GSE160403: DNA-seq of MLL-AF9 WT and MLL-AF9 *Mir139*KO cells transduced with LentiCRISPRv2 GeCKOv2 sgRNA library. The mass spectrometry proteomics data have been deposited to the ProteomeXchange Consortium via the PRIDE partner repository with the identifier PXD022488.
