# Supplementary material for: The tumor suppressor MIR139 is silenced by POLR2M to promote AML oncogenesis
Source: Leukemia. 2021 Nov 5;36(3):687–700. doi: 10.1038/s41375-021-01461-5 (PMC8885418; doi:10.1038/s41375-021-01461-5)
Supplement: Supplementary file 1 — Supplementary Information [file 41375_2021_1461_MOESM1_ESM.docx]

**Supplementary Methods**

**In vitro transcription of gRNAs**

All gRNAs (Supplementary Table 1) were designed according to the protocol of TrueCut Cas9 Protein v2 (A36499, Thermo Fisher Scientific). Subsequently, the DNA templates encoding the gRNAs are transcribed by the RNAMaxx High Yield Transcription Kit (200339, Agilent Technologies) according to manufacturer’s protocols. The quality and the size of the generated gRNAs was analyzed using the Agilent 2100 Bioanalyzer with a RNA6000 nano assay.

**Construction of lentiviral vectors**

DNA oligonucleotides containing sgRNA sequences (Integrated DNA Technologies) were annealed and cloned into LentiCRISPRv2 (Addgene #52961) as described previously ^1^. All oligonucleotides used are listed in Supplementary Table 1. The DOX inducible *miR-139* vector (pCW- i139) was generated by amplifying the *Mir139* gene with 200 bp flanking sequences using primers mmumiR139FW and mmumiR139RV listed in Supplementary Table 1. The DOX inducible eGFP vector (pCW-ieGFP) was generated by amplifying eGFP from pMSCV-IRES-eGFP using primers eGFPFW and eGFPRV listed in Supplementary Table 1. The amplicons were cloned into pENTR-D-TOPO (K243520, Thermo Fischer Scientific) according to manufacturer’s protocols and subsequently cloned into pCW57.1 (Addgene, 41393) by using Gateway LR Clonase II (11791020, Invitrogen) according to manufacturer’s protocol.

**Virus production**

Human embryonal kidney cells (HEK293FT, Thermo Fisher Scientific) or BOSC23 human kidney cells (a kind gift of the Dr. D.G Tenen lab) were cultured in Dulbecco’s Modified Eagle Medium (DMEM), supplemented with P/S (100 U/mL), Heat-Inactivated (HI) FBS (10%) (10270106, Thermo Fisher Scientific) and L-Glutamine (2 mM; 25030-024, Gibco Technologies). For the production of MSCV-MLL-AF9 viruses, HEK293FT cells were co-transfected with pCL-Eco and pMSCV neo-MLL-AF9 using Fugene 6 (E2691, Promega) or Lipofectamine 2000 (11668019, Thermo Fischer Scientific) according to manufacturer’s protocols. For the production of MSCV-MLL-AF9-GFP viruses, BOSC23 cells were transfected with pMSCV-MLL-AF9-GFP ^2^ using Lipofectamine 2000 (11668019, Thermo Fischer Scientific) according to manufacturer’s protocols. For lentivirus production, HEK293FT cells were co-transfected with lentiCRISPRv2 (Addgene, #52961), or pCW-*miR-139*, pVSVg (Addgene, #8454) and psPAX2 (Addgene, #12260) using Fugene 6. The Mouse CRISPR Gecko v2 pooled library (Addgene, #1000000052) was amplified and lentivirus was generated according to protocols ^1^. Culture supernatant containing retro- or lentiviruses were filtered (45µm) 48 hours post-transfection and subsequently used for transduction.

**Generation of MLL-AF9 and MLL-AF9-i139 cells**

HSPCs were isolated from femur and tibiae from WT, *Mir139*KO, E1KO or E2KO C57BL/6 mice (8 – 20 wks) using the BD Mouse Hematopoietic Progenitor (Stem) Cell Enrichment Set (cat 558451, Becton Dickinson). HPSCs were expanded in StemPro-34 SFM (Thermo Fisher Scientific, 10639011) supplemented with Interleukin (IL)-3 (1:1000 supernatant), IL-6 (10 ng/mL; SRP3330 Sigma Aldrich), Granulocyte-Macrophage Colony-Stimulating Factor (GM-CSF) (10 ng/mL; CYT-222, Prospec Ltd) Stem Cell Factor (SCF) (20 ng/mL; 5223SF, Cell Signaling Technology) and Penicillin-Streptomycin (P/S) (100U/mL; 15140163, Thermo Fisher Scientific) overnight and transduced with MSCV-MLL-AF9-neo or MSCV-MLL-AF9-puro retroviruses ^3^ using RetroNectin (T100A, Takara) according to manufacturer’s protocol. Transduced cells were selected with Neomycin (800µg/mL; 10131035, Thermo Fisher Scientific) or Puromycin (1µg/mL; P8833-25MG). After at least two re-platings in MethoCult (M3231, Stem Cell Technologies), MLL-AF9 cells were further expanded in Iscove’s Modified Dulbecco’ Media (IMDM) (12440053, Thermo Fisher Scientific), supplemented with P/S (100 U/mL), FBS (10%) (S181G-500, Biowest), IL-3 (1:1000 supernatant), IL-6 (10 ng/mL), GM-CSF (10 ng/mL) and SCF (20 ng/mL). For the generation of DOX-inducible *miR-139* cells (MLL-AF9-i139), MLL-AF9 cells were transduced with Babe-tetR-KRAB-Hygro retroviruses^4^ with RetroNectin according to manufacturer’s protocol and selected on Hygromicin B (0.8 mg/mL; 10687010, Thermo Fisher Scientific) for two weeks. Next, selected cells were lentivirally transduced with CW-*miR-139* viruses by spinfection (centrifugation of MLL-AF9-tetR-KRAB-Hygro cells at 800xg for 99 minutes at 37°C) and incubated in expansion medium and plated in MetchoCult with Puromycin (1 µg/mL; P8833, Sigma Aldrich) for 24 hours. To generate MLL-AF9-i139 cells, individual clones were picked and further expanded for experiments.

**Generation of MLL-AF9-GFP-TetR-KRAB-i139 chimeric mice**

BM cells were isolated from Pep boy B6 (CD45.1) mice and Lin- cKit+ Sca-1+ (LSK) cells were sorted using Influx instrument (BD Biosciences, San Jose, CA, USA). LSKs were cultured in StemSpam™ Serum-Free Expansion Medium (SFEM) (09650, StemCell Technologies) supplemented with IL-3 (10 ng/ml, 213-13, Peprotech), IL-6 (10 ng/mL, 216-16, Peprotech), TPO (25 ng/ml, 315-14, Peprotech), SCF (50 ng/mL, 250-03, Peprotech), FLT-3 ligand (50ng/mL, 250-31L, Peprotech). Subsequently, LSKs were retrovirally transduced with MSCV-MLL-AF9-GFP and this was repeated 24 hours post-transduction. After 48 hours, the percentage of transduced GFP^+^ MLL-AF9 cells was determined by flow cytometry. In total, 1000 MLL-AF9 GFP^+^ cells were transplanted via tail-vein injection into lethally-irradiated congenic mice (C57BL/6 CD45.2) together with 5x10^5^ BM support cells (C57BL/6 CD45.2). Recipient mice developed leukemia 4-5 weeks after transplantation.

Spleens were isolated from MLL-AF9 leukemic mice and cultured in StemSpam SFEM in the presence of IL-3 (10 ng/mL), IL-6 (10 ng/mL), and SCF (20 ng/mL). MLL-AF9-GFP cells were transduced with pBabe-TetR-KRAB retroviruses for two consecutive days, followed by a transduction with CW-*miR-139* lentiviruses the day after. After 48 hours, MLL-AF9 cells were seeded in semi-solid medium (M3231, StemCell Technologies) supplemented with IL-3 (10 ng/mL), IL-6 (10 ng/mL), and SCF (20 ng/mL), Puromycin (5 µg/mL, ant-pr-1, Invitrogen) and Hygromycin B (1.2 mg/mL, H3274, Sigma Aldrich). After 10 days, individual colonies were picked, and cell suspensions were replated in semi-solid medium supplemented with IL-3 (10 ng/mL), IL-6 (10 ng/mL), and SCF (20 ng/mL), Puromycin (5 µg/mL) and Hygromycin B (1.2 mg/mL) in the presence or absence of Doxycycline (5 µg/mL, D9891-10G, Sigma-Aldrich). DOX-responsive MLL-AF9-GFP-TetR-KRAB-i139 clones were harvested and transplanted into sublethally irradiated congenic mice (CD45.2) by tail vein injection. Recipient mice were sacrificed when moribund and BM cells and splenocytes were harvested and expanded *in vitro*. In total, 1 x 10^4^ MLL-AF9-GFP-TetR-KRAB-i139 splenocytes were retransplanted into sublethally irradiated recipients (CD45.2). Half of the recipient mice received doxycycline (4 mg/kg) in the drinking water supplemented with sucrose (10 g/L). Leukemic mice were sacrificed when moribund, and leukemia was confirmed by flow cytometry.

**Genome wide CRISPR-Cas9 sgRNA screen**

MLL-AF9 and MLL-AF9-139KO cells were transduced with the lentiCRISPR GeCKO v2 pooled library with a 500 fold coverage and 20 – 30% transduction efficiency. MLL-AF9 cells were harvested 24 hours after transduction. Puromycin (1µg/mL) was added to the medium for selection of the transduced cells. Cells were expanded for 14 days. DNA was isolated using the DNeasy Blood & Tissue Kit (Qiagen, 69506) according to manufacturer’s protocol, with the addition of 400 µg RNase A (Qiagen) per 5 million cells. To determine the complexity of the library in the cell populations, the lentiviral sgRNA constructs in the genomic DNA were amplified as previously described ^1^, with a few modifications. Briefly, the first PCR was performed for each sample in 13 separate 100 µL reactions. Each reaction contained 10 µg genomic DNA to get an estimated 300 times library representation using High Fidelity Herculase II Fusion DNA Polymerase (600675, Agilent Technologies), dNTPs (Invitrogen) and 0.5 µM of primers sgRNAlibF1 and sgRNAlibR1 (Supplementary Table 1). The sequences encoding the sgRNAs were amplified using the following PCR conditions: 2 min at 95°C, 20 cycles of (30 seconds at 95°C, 30 seconds at 55°C, 1 minute at 72°C) and fragments were extended for 10 min at 72°C. All PCR reactions were pooled and mixed. The nested PCR reaction was performed for each sample in 6 separate 100 µL reactions. Each reaction contained 5 µL of the first PCR reaction, Fusion polymerase, dNTPs and primers sgRNAlibF2 (0.5µM) and sgRNAlibR2 (0.5µM). The concentration and quality of the libraries were determined by Agilent 2100 Bioanalyzer using a DNA1000 assay.

Cluster generation was performed according to the Illumina TruSeq SR Rapid Cluster kit v2 (cBot) Reagents Preparation Guide (Illumina). Briefly, pooled libraries (10nM) were denatured with NaOH, diluted to 10 pM and hybridized onto the flowcell. The hybridized products were sequentially amplified, linearized and end-blocked according to the Illumina Single Read Multiplex Sequencing user guide (Illumina). After hybridization of a custom-made sequencing primer sgRNAlibseq (Supplementary Table 1), sequencing-by-synthesis was performed with the HiSeq 2500 (Illumina) using a single read 50-cycle protocol followed by single index sequencing.

**Genome-wide CRISPR-Cas9 sgRNA screen data analysis**

The mouse GeCKOv2 sgRNA library tables were converted to FastA format with the target gene and sgRNA identifier in the header. From this FastA file a HISAT2 index was constructed with HISAT2 (version 2.1.0) ^5^. Variations of the sgRNA vector sequence (GTTTTAGAGCT, GTTTAGAGCT and GTTTTTAGAGCT) were trimmed from the 3-prime ends of the reads using AdapterTrimmer (https://github.com/erasmus-center-for-biomics/AdapterTrimmer) with the following parameters: “--maximum-mismatches 2”, “--minimum-matches 2” and “--minimum-bases-remaining 15”. The reads were then aligned to the mouse GeCKOv2 library using HISAT2 (version 2.1.0) ^5^ with the “--no-spliced-alignment” and “--norc” parameters to make sure reads aligned without inserts and in the correct orientation. The resulting alignments were converted to sorted BAM files with SAMtools (version 1.10) ^6^. The number of reads per sgRNA was subsequently determined from the primary alignments by retrieving the reference sequence name using SAMtools view and awk, sorting the result and counting the number of occurrences using uniq with the “–c” parameter. The resulting table was converted to a comma-delimited file using sed and awk. Subsequent data analysis was performed in R (version 4.0.2). Statistical analysis was performed using the PBNPA package ^7^ on all guide RNAs with at least 11 reads in the experiment. General data handling and visualization was performed using the tidyverse package ^8^ and pipelining was handled using drake ^9^.

**Viability assays**

WT, *Mir139*KO, E1KO or E2KO MLL-AF9 cell*s* (n= 30,000) were plated in opaque plates and treated with different concentrations of UNC1999 or UNC2400 (4905/10, Tocris-Bioscience) in IMDM, supplemented with P/S (100 U/mL), FBS (10%), IL-3 (1:1000 supernatant), IL-6 (10 ng/mL), GM-CSF (10 ng/mL) and SCF (20 ng/mL). Medium was refreshed on a daily basis. Cell viability was determined with CellTiter-Glo Luminescent Cell Viability Assay (G7571, Promega) with the GloMax Explorer Multimode Microplate Reader (GM3500, Promega).

**Apoptosis assay**

50,000 MLL-AF9-i139 cells per clone were plated in MethoCult supplemented with P/S (100 U/mL), IL-3 (1:1000 supernatant), IL-6 (10 ng/mL), GM-CSF (10 ng/mL) and SCF (20 ng/mL), Hygromycin B (0.8 µg/mL), Puromycin (1 µg/mL) and with or without Doxycycline (5 µg/mL). MLL-AF9-i139 clones were harvested 24, 48, 72 or 96 hours after plating. Cells were washed with washing buffer (PBS, 0.5% BSA) and stained with the eBioscience Annexin V-FITC Apoptosis Detection Kit (BMS500FI-100, Invitrogen) according to manufacturer’s protocols.

**Flow Cytometry**

MLL-AF9 WT, MLL-AF9-*Mir139*KO, MLL-AF9-E1KO or MLL-AF9-E2KO cells were harvested and washed with FACS washing buffer (HBSS, FBS [3%], CaCl_2_ [1mM], Sodium azide [0.02%]) and stained with Annexin-V-APC (BD Biosciences, 550475) and antibodies against CD3 (CD3-PE-Cy7 BD Biosciences, 552774, Clone: 145-2C11), GR-1 (GR-1-AF700 Biolegend, 108422, Clone: RB6-8C5), CD11b (CD11b-APC-Cy7 BD Biosciences, 557657, Clone: M1/70), CD16/32 (CD16/32-PE Biolegend, 101308, Clone: 93), c-KIT (c-KIT-PE-Cy5 eBioscience, 15-1171-82, Clone: 2B8) in FACS washing buffer for 20 minutes at 4°C. To prevent non-specific binding, the cells were washed in FACS washing buffer and subsequently fixed with PFA (1%) with CaCl_2_ (1mM). These cells were measured on the BD LSRII-Fortessa (BD Biosciences). The data were processed using FlowJo VX (BD Biosciences).

**RNA sequencing**

HSPCs were isolated from WT and *Mir139*KO mice. Total RNA of WT HSPCs, *Mir139KO* HSPCs, MLL-AF9 cells and MLL-AF9-i139 cells was isolated with TRIzol, according to the manufacturer’s protocol. The quality of the RNA was analyzed with the Bioanalyzer RNA 6000 Nano Kit (5067-1511, Agilent). Libraries were generated with 500ng of RNA using the KAPA RNA HyperPrep Kit with RiboErase (KK8560, Roche) according to manufacturer’s protocol. Quality control of the libraries was performed with the Bioanalyzer High Sensitivity DNA kit (5067-4626, Agilent Technologies). Library quantity was determined with NEBNext Library Quant Kit (E6730, New England Biolabs). Libraries were run on the NovaSeq (Illumina). After sequencing and demultiplexing, the quality of the reads was determined with fastQC (v 0.11.8). Fastq files were mapped to the GRCm38 reference using STAR (v.2.5.3e). The annotation was done with FeatureCounts (v1.6.0) using genecodevM15.annotation.gtf as a reference. The quality of the data was assessed with MultiQC. Noise reduction was applied to the count data by removing the rows with read counts lower than 10. Next, rlog transformation was used for visualization and comparison purposes which include correlation and clustering analysis and the generation of the heatmaps and PCA plots. For differential expression analysis, DESeq2 (v1.22.2) was used.

**Luciferase reporter assay**

Oligo duplexes containing the predicted WT and mutated miR-139 binding motifs of the 3’-UTRs of *Hpgd*, *Ptprt* and *Eif4g2* (Supplementary Table 1) were cloned into the pmirGLO Dual-luciferase miRNA Target Expression Vector (E1330, Promega) with general cloning techniques. HEK293FT cells were co-transfected with pmirGLO vector and BCD2-MSCV-miR-139 (microRNA vector) or BCD4-MSCV-EV (control vector) in a molecular ratio of 1:15 with FuGENE6 Transfection Reagent (E2311, Promega). After 48h, Firefly and Renilla luciferase activities were measured by using the Dual-Glo luciferase Assay System (E2920, Promega) and the GloMax Explorer Multimode Microplate Reader (GM3500, Promega).

**Chromatin Immunoprecipitation and PCR**

Chromatin Immunoprecipitation (ChIP) assays were performed as previously described ^10^. Shortly, 10x10^6 Molm-13 cells were cross-linked in formaldehyde (1%) at RT for 10 min followed by quenching in a glycine solution (125 mM). Cells were lysed in nuclei lysis buffer (SDS [1%], Tris-HCl [50mM], EDTA [10 mM] and cOmplete (TM), EDTA-free Protease Inhibitors [11873580001, Sigma Aldrich) and sonicated with the Bioruptor Pico (B01060010, Diagenode) for 7 (Polr2m) or 9 (POL-II) cycles. Chromatin immunoprecipitation was performed with 2.5 µg antibodies against POLR2M (ABE23, Merck), RNA Polymerase II (POLII) CTD repeat (ab26721, Abcam) and IgG (31887, Thermo Fisher Scientific) at 4°C O/N. To determine POLR2M binding, qPCRs were performed with primers listed in Supplementary Table 1 and PowerUP SYBR Green Master Mix (ThermoFisher, A25742) according to manufacturer’s protocol.

**ChIP-seq analysis**

For analyzing publically available ChIP-seq datasets we used the Integrative Genomics Viewer v2.9.4. For the epigenetic analysis of the *Mir139* locus we used data deposited in the GEO database from ^11-13^. For the epigenetic analysis of the *MIR139* locus we used data deposited in the ENCODE database or UCSC Genome browser.

**Bisulfite conversion and sequencing**

Genomic DNA was isolated from AML cells with DNeasy Blood & Tissue Kit (Qiagen). Bisulfite conversion was performed with the EZ DNA Methylation-Gold Kit (D5005, Zymo Research), according to manufacturer’s protocols. The bisulfite and nested PCR primers (Supplementary Table 1) were designed using the Bisulfite Primer Seeker software by Zymo research and DNA fragments were amplified with Q5 High-Fidelity DNA Polymerase (NEB M0491S, New England Biolabs). PCR fragments were cloned in the CloneJET PCR Cloning Kit (K1231, Thermo Fisher Scientific), Sanger sequenced with the BigDye Terminator v3.1 Cycle Sequencing Kit (4337454, Thermo Fisher Scientific) and with the BigDye XTerminator Purification Kit (4375486, Thermo Fisher Scientific). PCR products were subsequently run on an ABI Prism 3130xl genetic analyzer (4359571, Applied Biosystems) or a 3500 Genetic Analyzer (4405673, Applied Biosystems) according to manufacturer’s protocols.

**Proteomics**
Cell lysis, digestion and TMT labeling was performed as described ^14^. In short, one million mouse MLL-AF9 cells or HSPCs were lysed in SDS lysis buffer (SDS [5%], Tris-HCl [100 mM, pH 7.6]) at 95°C for 4 minutes. The protein concentration per sample was determined by Pierce BCA protein assay (Thermo Fisher Scientific). 100 µg of protein was used for subsequent reduction with TCEP (5 mM), alkylation with iodoacetamide (15 mM) and quenching with DTT (10 mM). Protein lysates were purified by methanol-chloroform precipitation. The resulting protein pellets were resuspended in HEPES (40 mM, pH 8.4) and digested with Trypsin (10 µg) at 37°C O/N. Peptide concentration was measured with Pierce BCA assay.

A total of 10 µg of each of the 8 peptide preparations was dissolved in 25 µL of HEPES (40 mM, pH 8.4) and incubated with 40 µg of one of the 8 amino reactive TMT10plex Label Reagents (Thermo Fisher Scientific) at RT for 1h. Excess TMT label was quenched by incubation with 6 μL hydroxylamine (5%) at RT for 15 min. The 8 labelled peptide samples were then mixed, freeze-dried and measured using multinotch MS3.

TMT-labeled peptides were dissolved in water/formic acid (100/0.1 v/v) and subsequently analyzed by on-line C18 nanoHPLC MS/MS with a system consisting of an Easy nLC 1200 gradient HPLC system (Thermo, Bremen, Germany), and an Orbitrap Fusion LUMOS mass spectrometer (Thermo). Fractions were injected onto a homemade precolumn (100 μm × 15 mm; Reprosil-Pur C18-AQ 3 μm, Dr. Maisch, Ammerbuch, Germany) and eluted via a homemade analytical nano-HPLC column (50 cm × 75 μm; Reprosil-Pur C18-AQ 1.9 µm). The analytical column temperature was maintained at 50°C with a Sonation PRSO-V2 column oven. The gradient was run from 5% to 30% solvent B (water/acetonitrile/formic acid (FA) [20/80/0.1] v/v) in 240 minutes. The nano-HPLC column was drawn to a tip of ∼5 μm and acted as the electrospray needle of the MS source. The LUMOS mass spectrometer was set to use the multi-notch MS3-based TMT method ^15^. The MS1 spectrum was recorded in the orbitrap (resolution 120,000; mass range 400−1500 m/z; automatic gain control (AGC) target 2 × 10^5^; maximum injection time 50 ms). Dynamic exclusion was after n=1 with an exclusion duration of 60 s with a mass tolerance of 10 ppm. Charge states 2-4 were included. Precursors for MS2/MS3 analysis were selected using a TopSpeed of 3 seconds. MS2 analysis consisted of collision-induced dissociation (quadrupole ion trap analysis; AGC 1 × 10^4^; normalized collision energy (NCE) 35; maximum injection time 50 ms). The isolation window for MS/MS was 0.7 Da. Following acquisition of each MS2 spectrum, the multinotch MS3 spectrum was recorded using an isolation window for MS3 of 2 Da. MS3 precursors were fragmented by high energy collision-induced dissociation (HCD) and analyzed using the Orbitrap (NCE 65; AGC 1 × 10^5^; maximum injection time 105 ms, resolution 60,000).

In a post-analysis process, raw data were first converted to peak lists using Proteome Discoverer version 2.2 (Thermo Electron) and submitted to the Uniprot database (*Mus musculus*, 52015 entries), using Mascot v. 2.2.07 (www.matrixscience.com) for protein identification. Mascot searches were with 10 ppm and 0.02 Da deviation for precursor and fragment mass, respectively, and trypsin as enzyme. Up to two missed cleavages were allowed, and methionine oxidation was set as a variable modification; carbamidomethyl on Cys, TMT6plex on N-term and Lys were set as a fixed modification. The protein FDR was set to 1%.

**RNA isolation and RT-PCR**

Total RNA was isolated with TRIzol (15596026, Thermo Fisher Scientific) according to manufacturer’s protocol. For miRNA expression analysis, cDNA was synthesized with the Taqman MicroRNA reverse transcription kit (Thermo Fisher Scientific) for *hsa-miR-139-3p* (002313, Thermo Fisher Scientific), hsa-*miR-139*-5p (002289, Thermo Fisher Scientific) and snoRNA U6 (001973, Thermo Fisher Scientific). The murine and human *miR-139-5p* and *miR-139-3p* are identical. For determination of *Pde2a* (Mm01136644_m1 and Hs00159935_m1, Thermo Fisher Scientific), *Cdkn2a* (Mm00494449_m1, Thermo Fisher Scientific), *Polr2m* (Mm0072465_m1 and Hs00930169_g1, Thermo Fisher Scientific) and *Gapdh* (Mm99999915_g1 and Hs02786624_g1, Thermo Fisher Scientific), RNA was reversely transcribed using the High-Capacity cDNA Reverse Transcription Kit (4368814, Thermo Fisher Scientific). For the quantification of human *pri-miR-139* in human patient samples*,* cells were treated with apoptosis inhibitor Q-VD-OPH (20µM, SML0063-1MG, Sigma-Aldrich) for 48 hours to prevent loss of *miR-139*-inducing cells. Total RNA of Molm-13 and patient samples were transcribed using SuperScript VILO cDNA Synthesis Kit (11754050, Invitrogen) according to manufacturer’s protocols. The *pri-miR-139* was pre-amplified using primerset TSS_miR139_UNC. Subsequently, the pre-amplified product was subjected to quantative PCR using primerset TSS_miR139_NESTED and the PowerUP SYBR Green Master Mix (ThermoFisher, A25742) according to manufacturer’s protocol. Gene and miRNA expression levels were detected with Taqman Gene Expression Assays (Thermo Fisher Scientific) on the QuantStudio™ 5 Real-Time PCR System (A28138, Thermo Fisher Scientific). Relative expression was calculated with the 2^ΔΔCT^ method or normalized against the input (*pri-miR-139*).

**References**

1. Sanjana NE, Shalem O, Zhang F. Improved vectors and genome-wide libraries for CRISPR screening. *Nat Methods* 2014 Aug; **11**(8)**:** 783-784.

2. Krivtsov AV, Twomey D, Feng Z, Stubbs MC, Wang Y, Faber J*, et al.* Transformation from committed progenitor to leukaemia stem cell initiated by MLL-AF9. *Nature* 2006 Aug 17; **442**(7104)**:** 818-822.

3. Wong P, Iwasaki M, Somervaille TC, So CW, Cleary ML. Meis1 is an essential and rate-limiting regulator of MLL leukemia stem cell potential. *Genes Dev* 2007 Nov 1; **21**(21)**:** 2762-2774.

4. Kumar MS, Erkeland SJ, Pester RE, Chen CY, Ebert MS, Sharp PA*, et al.* Suppression of non-small cell lung tumor development by the let-7 microRNA family. *Proc Natl Acad Sci U S A* 2008 Mar 11; **105**(10)**:** 3903-3908.

5. Kim D, Paggi JM, Park C, Bennett C, Salzberg SL. Graph-based genome alignment and genotyping with HISAT2 and HISAT-genotype. *Nat Biotechnol* 2019 Aug; **37**(8)**:** 907-915.

6. Li H, Handsaker B, Wysoker A, Fennell T, Ruan J, Homer N*, et al.* The Sequence Alignment/Map format and SAMtools. *Bioinformatics* 2009 Aug 15; **25**(16)**:** 2078-2079.

7. Jia G, Wang X, Xiao G. A permutation-based non-parametric analysis of CRISPR screen data. *BMC Genomics* 2017 Jul 19; **18**(1)**:** 545.

8. Wickham H*ea*. Welcome to the {tidyverse}. *J Open Source Softw* 2019; **4**(43)**:** 1686.

9. Landau WM. The drake R package: a pipeline toolkit for reproducibility and high-performance computing. *J Open Source Softw* 2018; **3**(21).

10. Bindels EM, Havermans M, Lugthart S, Erpelinck C, Wocjtowicz E, Krivtsov AV*, et al.* EVI1 is critical for the pathogenesis of a subset of MLL-AF9-rearranged AMLs. *Blood* 2012 Jun 14; **119**(24)**:** 5838-5849.

11. Lara-Astiaso D, Weiner A, Lorenzo-Vivas E, Zaretsky I, Jaitin DA, David E*, et al.* Immunogenetics. Chromatin state dynamics during blood formation. *Science* 2014 Aug 22; **345**(6199)**:** 943-949.

12. Volk A, Liang K, Suraneni P, Li X, Zhao J, Bulic M*, et al.* A CHAF1B-Dependent Molecular Switch in Hematopoiesis and Leukemia Pathogenesis. *Cancer Cell* 2018 Nov 12; **34**(5)**:** 707-723 e707.

13. Xu B, On DM, Ma A, Parton T, Konze KD, Pattenden SG*, et al.* Selective inhibition of EZH2 and EZH1 enzymatic activity by a small molecule suppresses MLL-rearranged leukemia. *Blood* 2015 Jan 8; **125**(2)**:** 346-357.

14. Paulo JA, Gygi SP. Nicotine-induced protein expression profiling reveals mutually altered proteins across four human cell lines. *Proteomics* 2017 Jan; **17**(1-2).

15. McAlister GC, Nusinow DP, Jedrychowski MP, Wuhr M, Huttlin EL, Erickson BK*, et al.* MultiNotch MS3 enables accurate, sensitive, and multiplexed detection of differential expression across cancer cell line proteomes. *Anal Chem* 2014 Jul 15; **86**(14)**:** 7150-7158.

**Supplementary Figure Legends**

**Supplementary Fig. 1: MLL-AF9 downregulates *Mir139* expression.** (A) The number of Colony Forming Units (CFU) per 5000 MLL-AF9 cells treated with indicated concentrations of DOT1L-inhibitor SGC0946 is shown. (B) Volcano plot indicating the log2 ratio of protein levels in MLL-AF9 (MA9) cells relative to WT cells (X-axis) versus the -log10 P-value is depicted. Green rectangle indicates significantly downregulated proteins in MLL-AF9 cells, red rectangle indicates significantly upregulated proteins. Red circle around data point indicates HPGD. (C) Bar chart showing the –log10 P-values of the Diseases and Bio Functions, generated by the Ingenuity Pathway Analysis of the proteins shown in B. The Fisher’s exact test was used for the statistical analysis. (D) Gene Set Enrichment Analysis (GSEA) plot showing enrichment of MLL-AF9 targets in the upregulated fraction in MLL-AF9 cells (MLL) compared to WT cells. The Normalized Enrichment Score (NES), False Discovery Rate (FDR) Family Wise Error Rate (FWER) are shown below the GSEA plot. (E) Expression of *Cebpa*, *Csf1r*, *Csf3r*, and *Id2* in normalized ^2^log(counts) in MLL-AF9 cells (n=6) and WT HSPCs (n=4) cells as determined by RNA-seq. The Wald’s test with Benjamin Hochberg correction was used for statistical analysis. (F) GSEA plot showing enrichment of PRC1, EZH2, EZH2-controlled and EZH2-uncontrolled PcG targets, in the downregulated fraction of MLL-AF9 cells compared to WT HSPCs. All graphs are depicted as mean ± SEM.

**Supplementary Fig. 2:** ***MiR-139* upregulation and repression of *miR-139* targets eliminates MLL-AF9 AML *in vitro* and inhibits leukemogenesis in mice.** (A) Volcano plot showing the log2 ratio of protein abundances in WT HSPC relative to *Mir139*KO HSPCs (X-axis) versus the –log10 P-value (Y-axis). Green rectangle indicates significantly downregulated proteins, red rectangle indicates significantly upregulated proteins. (B) Cell counts of DOX treated LSK-MLL-AF9-i139 (n=7 clones) cells relative to non-treated cells are shown. The Wilcoxon matched-pairs signed rank test was used for the statistical analysis. (C) Flow cytometric analysis of BM isolated from WT or MLL-AF9 leukemic mice. *miR-139* expression was modulated by the presence (+ DOX) or absence (- DOX) of DOX in the drinking water. (D) Heatmap showing differentially expressed genes in MLL-AF9-i139 clones (n=3) that are treated with Doxycycline (+DOX; 5 µg/mL) compared to non-treated cells. (E) Schematic illustrating the genome-wide CRISPR/Cas9 KO screen strategy. MLL-AF9 cells are transduced with viruses expressing the sgRNA library followed by isolation of genomic DNA (gDNA) 24 hours post-transduction. Next, the MLL-AF9 cells are selected on Puromycin for 14 days followed by isolation of gDNA. Finally, the sgRNAs are quantified by next generation sequencing. (F) Fold change of expression of genes associated with cell cycle: *Btg2* (Padj: 0.00855), *Cdkn1a* (Padj: 0.00097) and *Cdkn2d* (Padj: 0.00140) or genes associated with apoptosis: *Bmf* (Padj: 0.00384), *Dusp1* (Padj: 0.00130)), *Rnasel* (Padj: 0.01690)), *Trp53inp1* (Padj: 0.00183), *Ypel3* (Padj: 0.00506) and *Zc3h12a* (Padj: 0.00079) in DOX treated (+DOX) MLL-AF9-i139 (n=3) clones (red) compared to non-treated (- DOX) cells (n=3, black) is shown. The Wald’s test with Benjamin Hochberg correction was used for the statistical analysis. (G) Colony Forming Units (CFU) of MLL-AF9 cells transduced with CRISPR-Cas9 sgRNAs control viruses (P) or CIRSPR-Cas9 sgRNA viruses targeting *Hpgd*, *Eif4g2* and *Ptprt* relative to the control sgRNAs (P) are shown. The two-tailed unpaired student’s T-test with Welch’s correction was used for the statistical analysis. The graph shows pooled data from two experiments in quadruple. (H) The ratio of Firefly Luciferase relative to Renilla Luciferase and to the mutated *miR-139* binding site (M) of indicated *miR-139* targets are depicted. The two-tailed unpaired student’s T-test was used for statistical analysis. Graphs show representative data of the experiments performed in quintuple.

**Supplementary Fig. 3: *Mir139* is epigenetically silenced by PRC2 in MLL-AF9 cells.** (A) The number of colony forming units (CFU) per 5000 WT or *Mir139*KO MLL-AF9 cells treated Trichostatin a (TSA) or Valporic acid (VPA) relative to untreated cells is shown. Presented data are representative of two experiments. (B) Expression levels of *miR-139-3p* and *miR-139-5p* in MLL-AF9 cells treated with indicated concentrations of UNC2400 relative to snRNA U6 and mock-treated MLL-AF9 cells are shown. (C) Viability of MLL-AF9 WT or MLL-AF9 *Mir139*KO cells treated with UNC2400 for 96 hours relative to mock-treated cells are shown. (D) Expression of *miR-139-5p* in untransformed WT HSPCs treated with UNC1999 relative to untreated cells and snRNA U6. Presented data of three mice are pooled. (E) *Cdkn2a* in MLL-AF9 cells treated with UNC1999 relative to *Gapdh* and mock-treated cells is depicted. (F) Expression of *Pde2a* in MLL-AF9 cells treated with indicated concentrations of UNC1999 relative to *Gapdh* and mock-treated cells is shown. Data are representative of three experiments. (G) Viability of THP-1 cells treated with indicated concentrations of UNC1999 relative to untreated cells is shown. The two-tailed paired student’s T–test was used for the statistical analysis. Data are representative of three experiments. (H) Expression of *miR-139-3p* and *miR-139-5p* relative to snRNA U6 and mock-treated cells in THP-1 cells treated with indicated concentrations of UNC1999. Data are representative of three experiments. (I) Viability of Molm-13, HL-60, MV4-11, HEL, Kasumi-1, TF-1 and U937 AML cell lines treated with UNC1999 relative to untreated cells is shown. The presented data are representative of three experiments. The asterisks indicate statistical significance. (J) Expression levels of *miR-139-5p* and *miR-139-3p* in Molm-13, HL-60, MV4-11, HEL, Kasumi-1, TF-1 and U937 AML cell lines treated with UNC1999 relative to snRNA U6 and mock-treated cells are shown. Presented data are representative for three experiments. The two-tailed paired student’s T-test was used for the statistical analysis in G and I. The two-tailed unpaired student’s T-test with Welch’s correction was used in A, C, E, F, H and J All graphs are depicted as mean ± SEM.

**Supplementary Fig. 4: POLR2M silences *Mir139* in MLL-AF9 cells.** (A) Volcano plot indicating –log10 P-values (Y-axis) and log2 fold change (X-axis) of all targeted genes in MLL-AF9 WT cells relative to MLL-AF9-*Mir139*KO cells. The six different plots indicate the number of sgRNAs per gene that were found with ≥ 10 counts in MLL-AF9 WT and MLL-AF9 *Mir139*KO cells. The blue marked circles are genes with at least 5 sgRNAs and a fold change ≥ 10 in the *Mir139*KO MLL-AF9 cells relative to the WT MLL-AF9 cells. The red closed circle indicates *Polr2m*. Red dashed lines indicate a fold change of ±10. (B) Expression levels of *miR-139-5p* and *miR-139-3p* in *POLR2M*-depleted Molm-13 cells relative to control targeted cells and snRNA U6 are shown. The two-tailed unpaired student’s T-test with Welch’s correction was used for statistical analysis. (C) Expression levels of *miR-139-5p* (left panel) and *miR-139-3p* (right panel) in *POLR2M*-depleted MLL-AF9 patient samples relative to control targeted cells and snRNA U6 are shown. The two-tailed unpaired student’s T-test with Welch’s correction was used for statistical analysis. (D) Expression of *Pde2a* in MLL-AF9 cells expressing sgRNAs targeting *Polr2m* (n=3) and control sgRNAs (n=3) relative to *Gapdh* is shown. The two-tailed unpaired student’s T-test with Welch’s correction was used for statistical analysis. (E) Expression levels of *miR-139-5p* (left panel) and *miR-139-3p* (right panel) in *POLR2M*-targeted U-937, MV4-11, TF-1 and HL-60 cells relative to control targeted cells and snRNA U6 are shown. The two-tailed unpaired student’s T-test with Welch’s correction was used for statistical analysis.

**Supplementary Fig. 5: POLR2M binds to E1, E2 and the TSS**. (A) ChIP-seq reads of the *PDE2A* locus (chr11:72,605,000 - 72,644,500) as determined in Human Umbilical Vein Endothelial Cells (HUVECs) and K562 human AML cells are depicted. The tracks show H3K4me3 signals (red), and H3K27Ac signals (orange) and POL-II binding (purple), per cell type. (B) Snapshot from the UCSC genome browser of the *PDE2A* locus (chr11:72,613,144-72,678,664), with the *MIR139* encoding sequence in green. Enrichment of histone marks H3K4Me1, H3K4Me3 and H3K27Ac are depicted in blue. GeneHancer identified promoter region (red) and enhancer regions (grey) are depicted below. The interactions of different elements are depicted in purple.

**Supplementary Table Legends**

**Supplementary Tables**

**Supplementary Table 1:** Excel file that contains the names and sequences of the primers used in the manuscript. Please find the data in the file: “Supplementary Table 1 Primersheet.xlsx”.

**Supplementary Table 2:** Excel file that contains the tables with RNA-sequencing data and Proteomics data of WT HSPCs vs MLL-AF9 cells. Please find the tables in the file: “Supplementary Table 2 WTvsMA9.xlsx”.

**Supplementary Table 3:** Excel file that contains the tables with the RNA-sequencing data and Proteomics data of WT HSPCs vs *Mir139*KO HSPCs. Please find the tables in the file: “Supplementary Table 3 WTvsKO.xlsx”.

**Supplementary Table 4:** Excel file that contains the tables with the RNA-sequencing data of MLL-AF9-i139 cells treated with or without DOX. Please find the tables in the file: “Supplementary Table 4 DOXvsNODOX.xlsx”.

**Supplementary Table 5:** Excel file that contains the tables with the genome-wide CRISPR-KO data of MLL-AF9 cells incubated for 24 hours and 14 days. Please find the tables in the file: “Supplementary Table 5 WT24hvsWT14d.xlsx”.

**Supplementary Table 6:** Excel file that contains the tables with the genome-wide CRISPR-KO data of MLL-AF9 cells vs MLL-AF9-*Mir139*KO cells incubated for 14 days. Please find the tables in the file: “Supplementary Table 6 KO14dvsWT14d.xlsx”.
