# Supplementary material for: The tumor suppressor MIR139 is silenced by POLR2M to promote AML oncogenesis
Source: Leukemia. 2021 Nov 5;36(3):687–700. doi: 10.1038/s41375-021-01461-5 (PMC8885418; doi:10.1038/s41375-021-01461-5)

Supplementary Figure 1

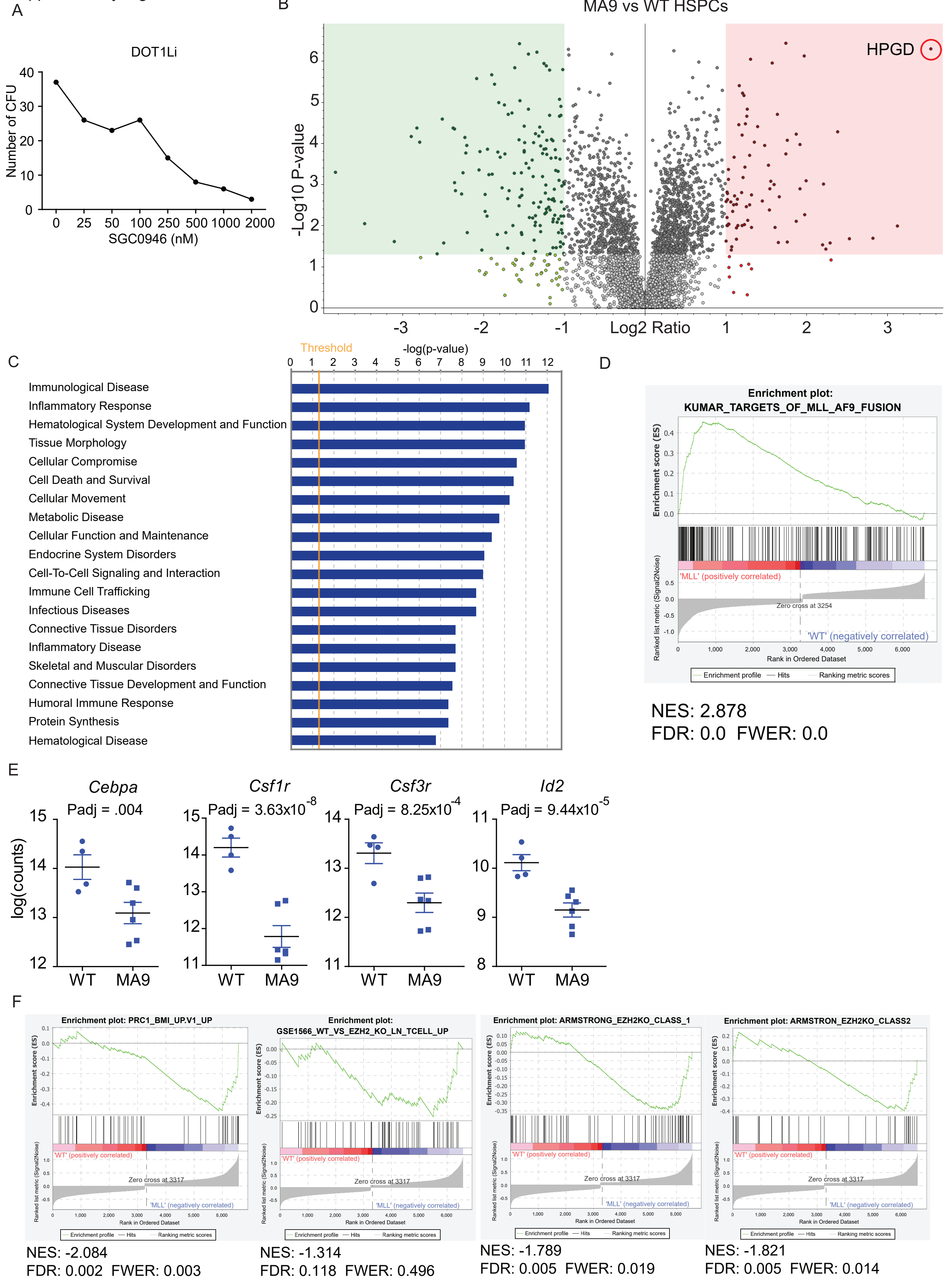

Supplement: Supplementary file 2 — Supplementary Figure 1 [file 41375_2021_1461_MOESM2_ESM.pdf]
