# Supplementary figures and images for: The tumor suppressor MIR139 is silenced by POLR2M to promote AML oncogenesis
Source: Leukemia. 2021 Nov 5;36(3):687–700. doi: 10.1038/s41375-021-01461-5 (PMC8885418; doi:10.1038/s41375-021-01461-5)

Supplementary Figure 2

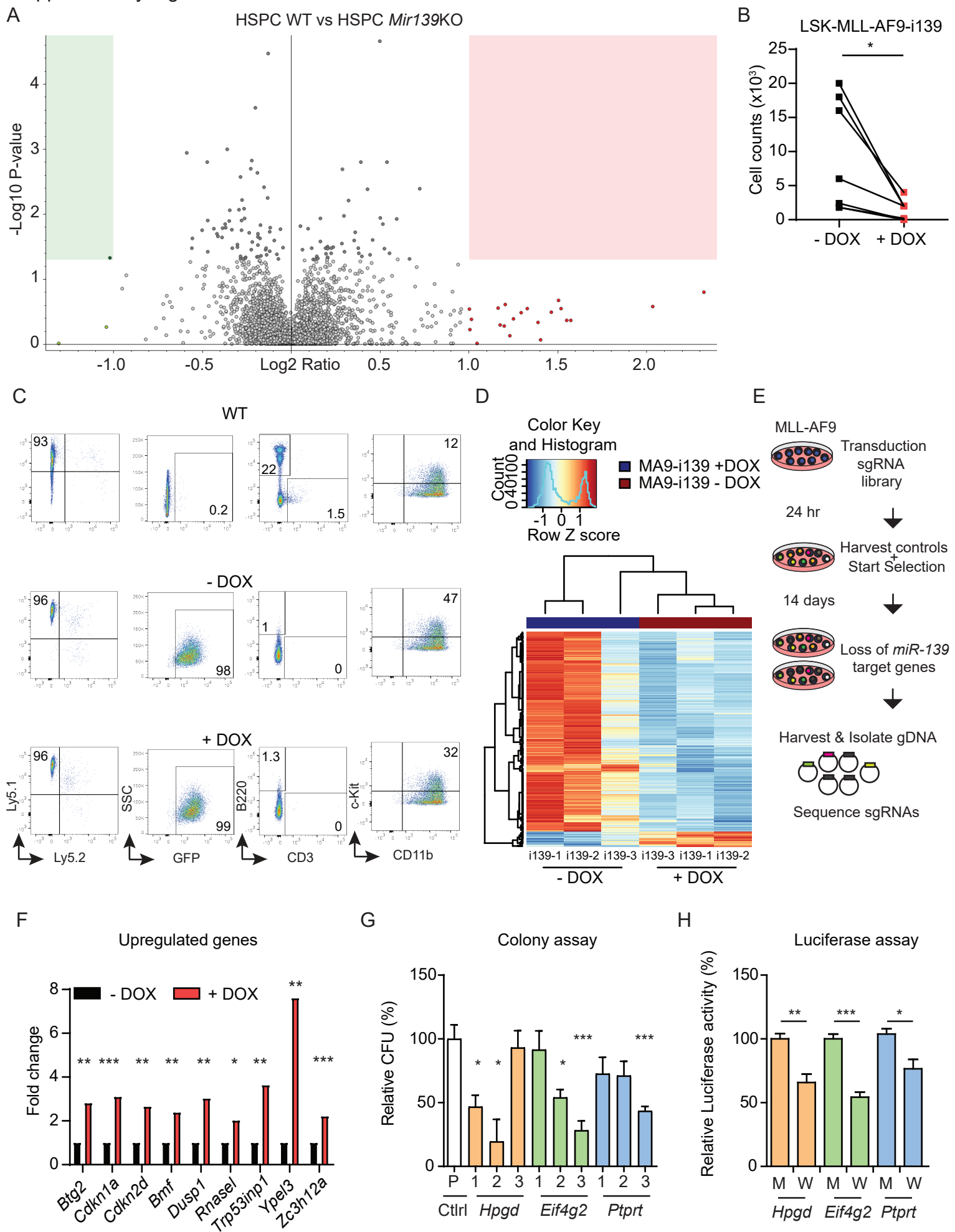

Supplement: Supplementary file 3 — Supplementary Figure 2 [file 41375_2021_1461_MOESM3_ESM.pdf]

Supplementary Figure 3

A

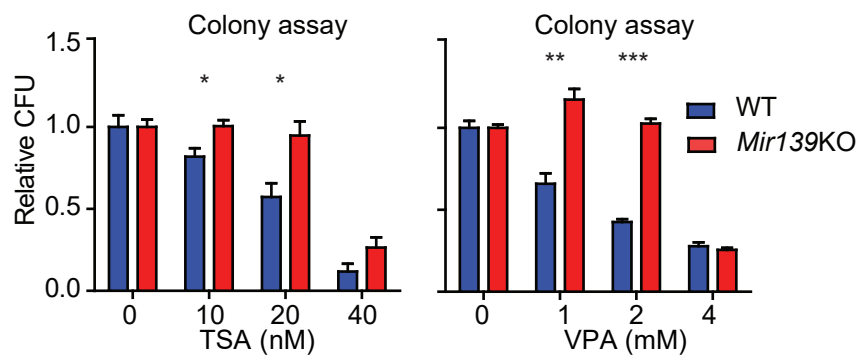

B

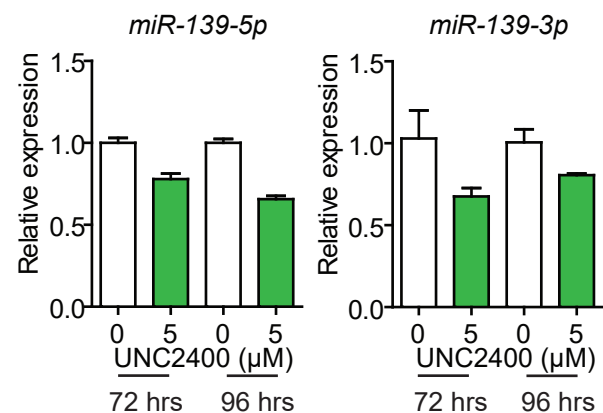

C

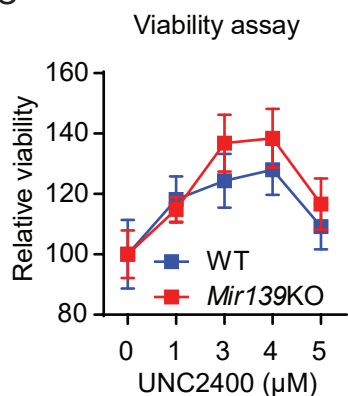

D

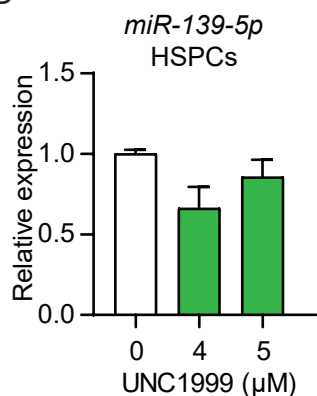

E

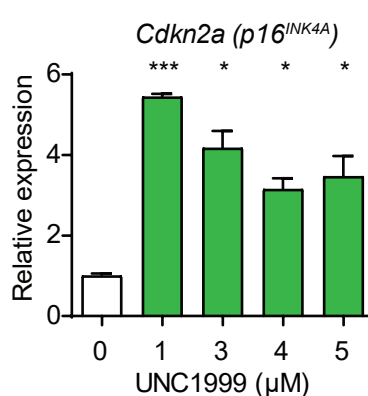

F

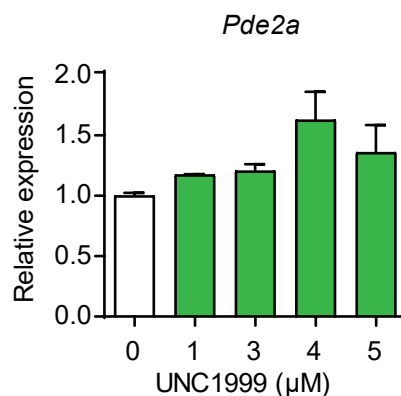

G

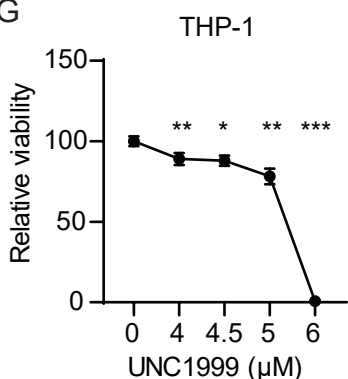

H

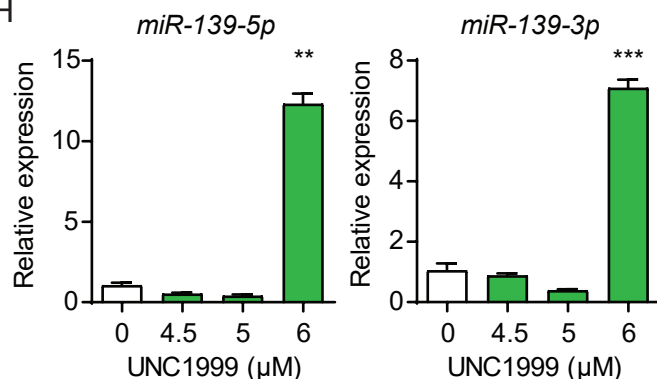

I

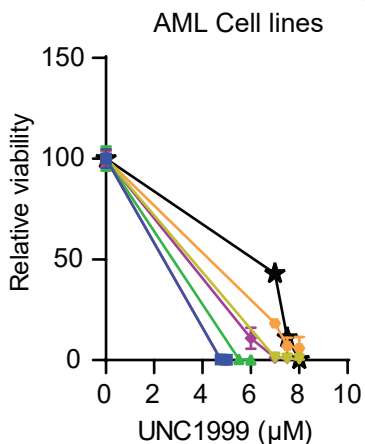

J

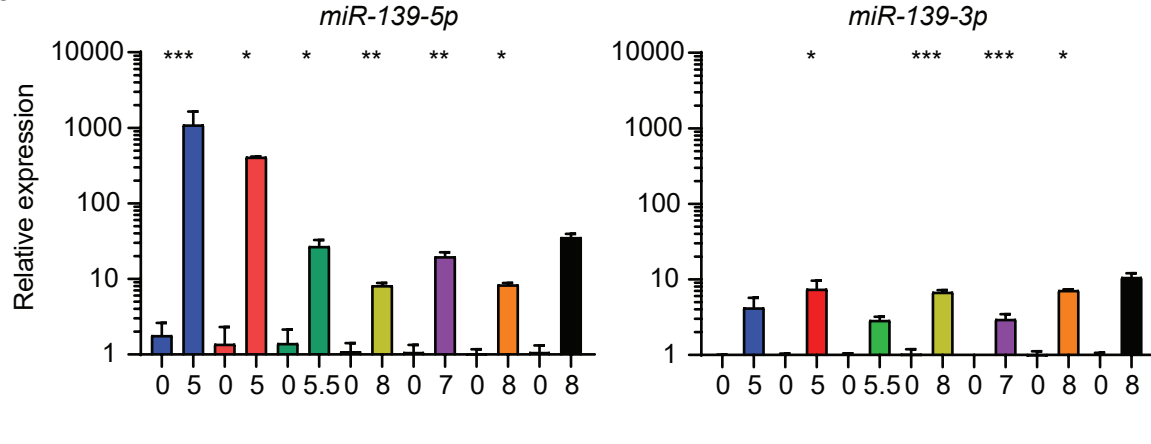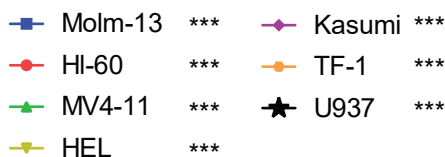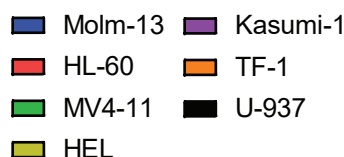

Supplement: Supplementary file 4 — Supplementary Figure 3 [file 41375_2021_1461_MOESM4_ESM.pdf]

Supplementary Figure 4

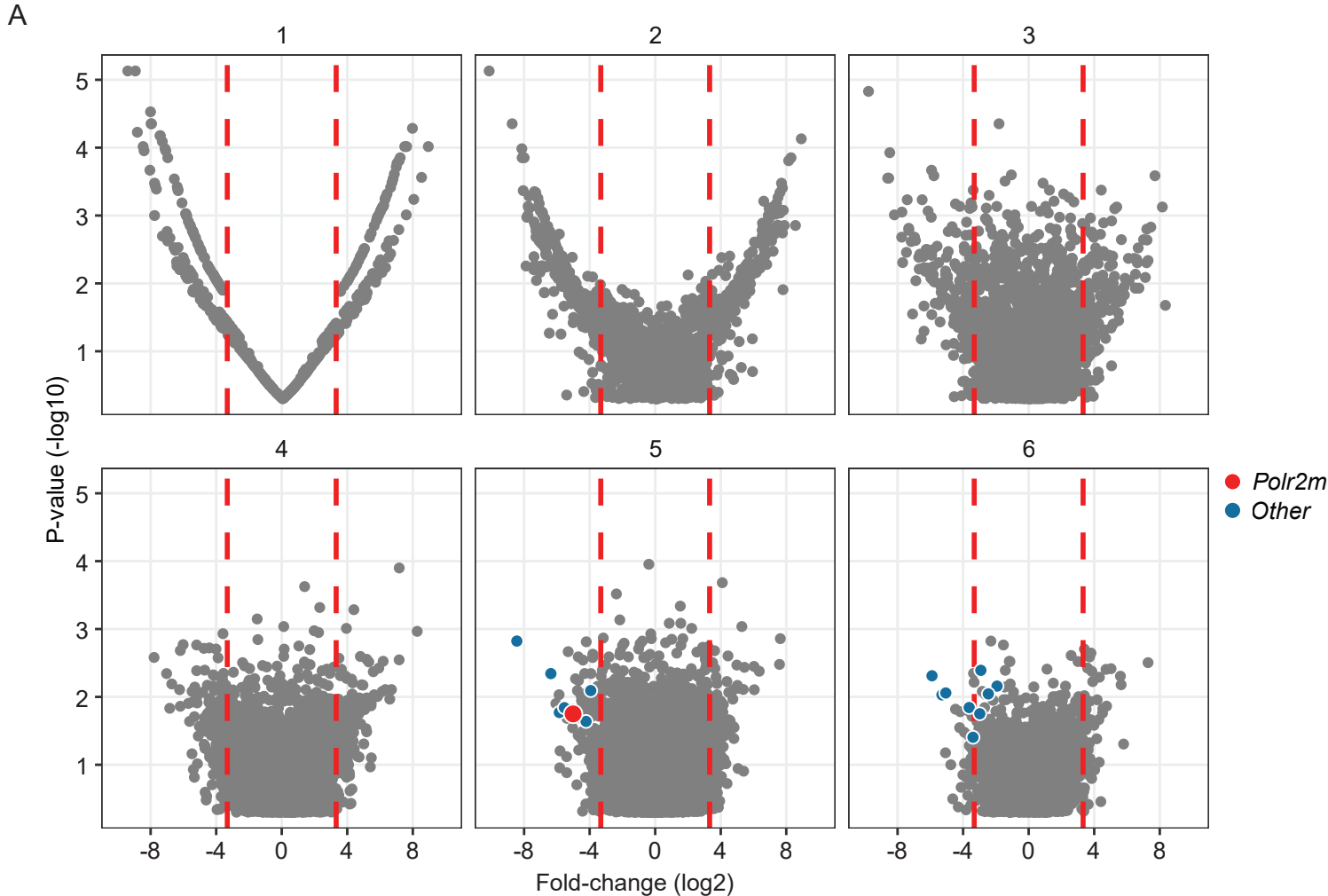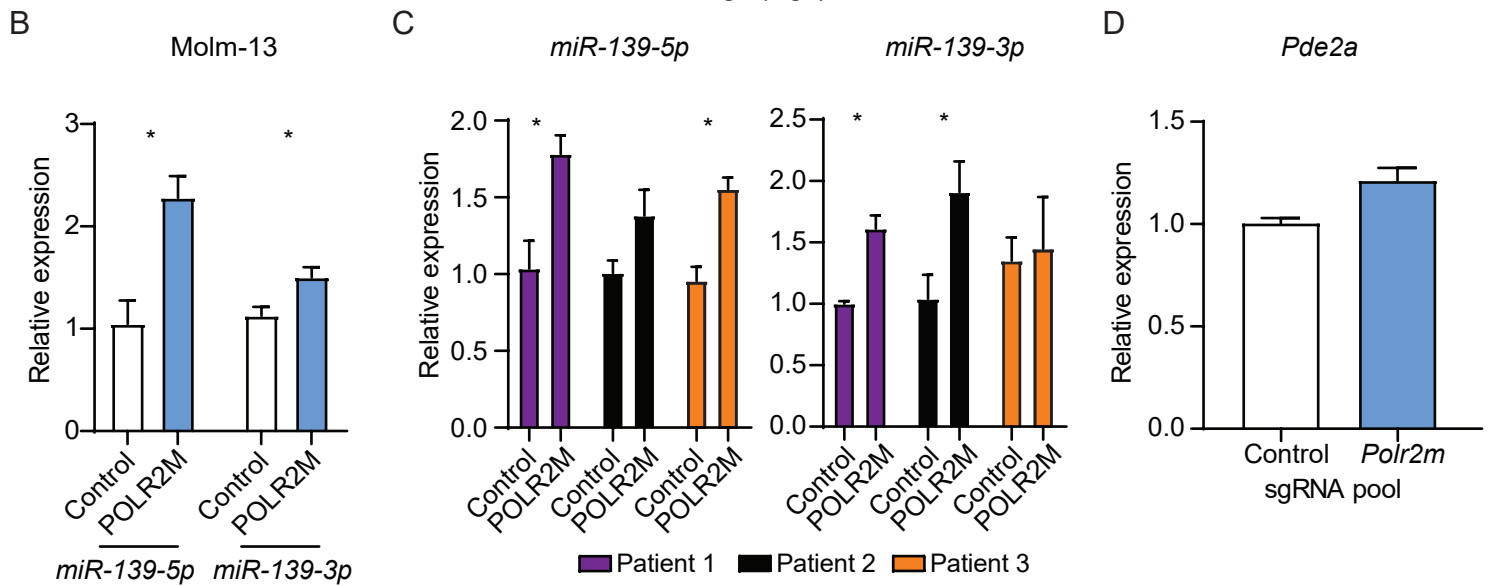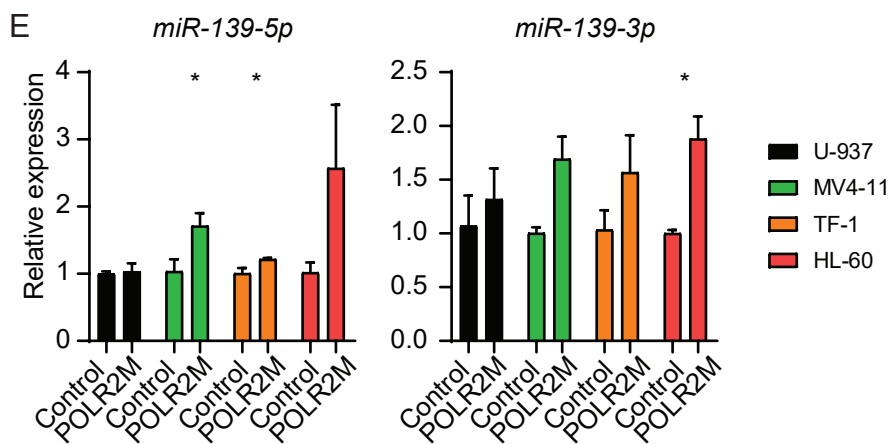

Supplement: Supplementary file 5 — Supplementary Figure 4 [file 41375_2021_1461_MOESM5_ESM.pdf]

Supplementary Figure 5

A

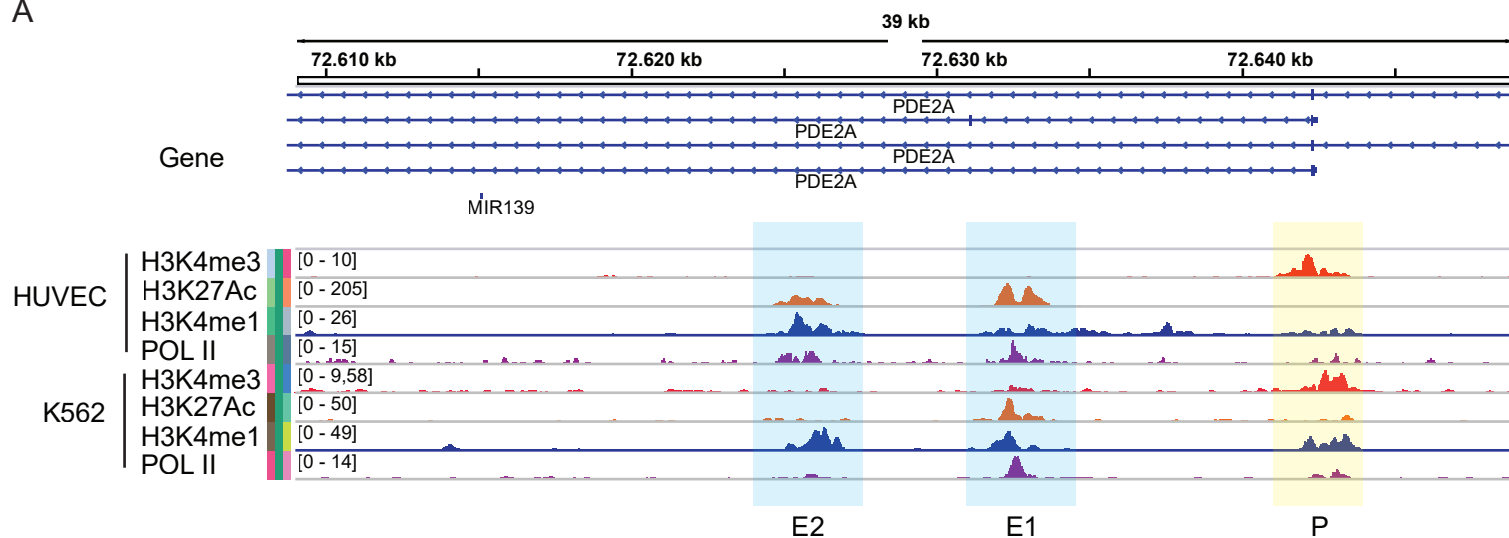

B

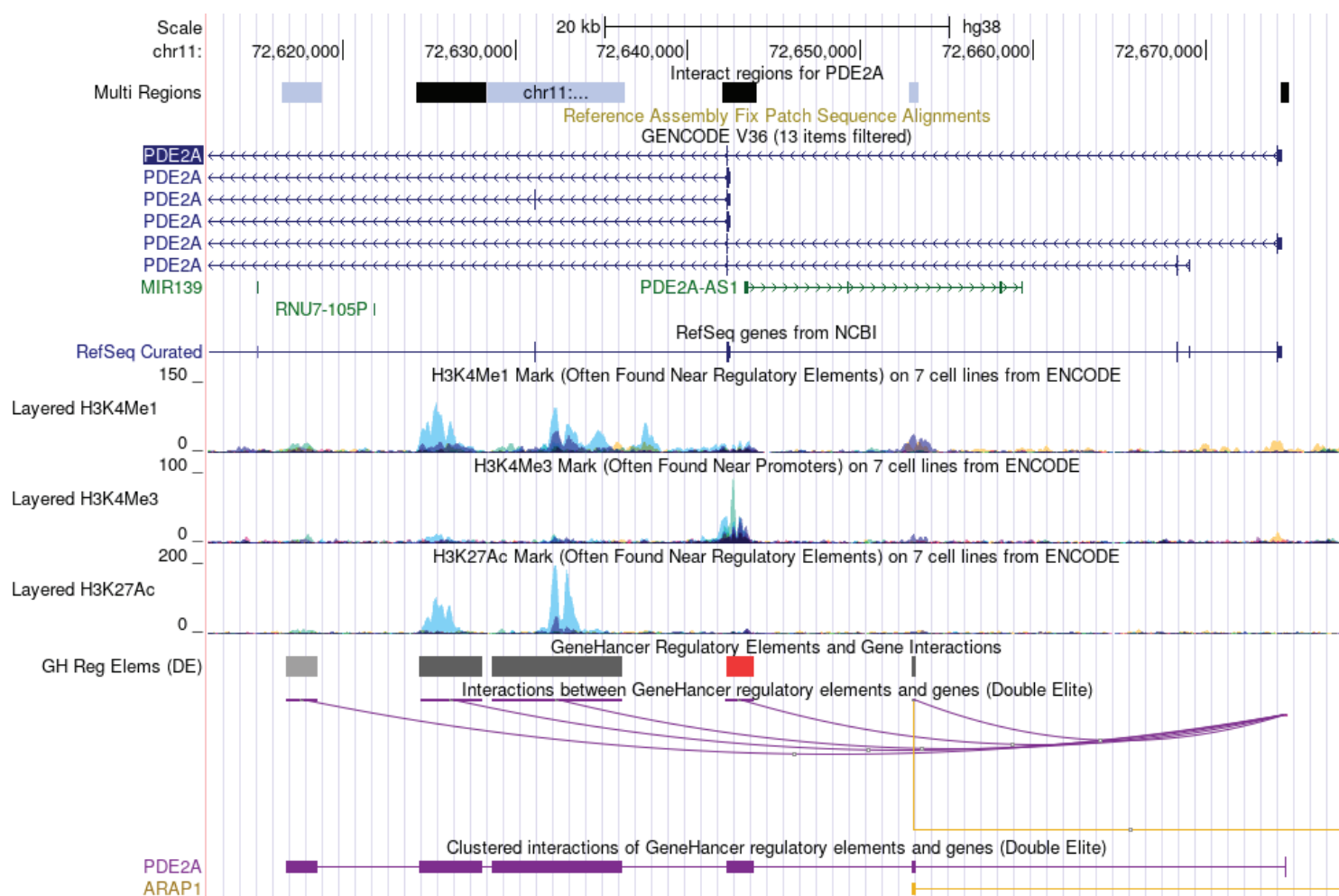

Supplement: Supplementary file 6 — Supplementary Figure 5 [file 41375_2021_1461_MOESM6_ESM.pdf]
